# Ischemic stroke disrupts the endothelial glycocalyx through activation of proHPSE via acrolein exposure

**DOI:** 10.1074/jbc.RA120.015105

**Published:** 2021-01-13

**Authors:** Kenta Ko, Takehiro Suzuki, Ryota Ishikawa, Natsuko Hattori, Risako Ito, Kenta Umehara, Tomomi Furihata, Naoshi Dohmae, Robert J. Linhardt, Kazuei Igarashi, Toshihiko Toida, Kyohei Higashi

**Affiliations:** 1 Graduate School of Pharmaceutical Sciences, Chiba University, Chiba, Japan; 2 RIKEN Center for Sustainable Resource Science, Wako, Japan; 3 Faculty of Pharmaceutical Sciences, Tokyo University of Science, Noda, Japan; 4 School of Pharmacy, Tokyo University of Pharmacy and Life Sciences, Tokyo, Japan; 5 Center for Biotechnology and Interdisciplinary Studies, Rensselaer Polytechnic Institute, Troy, New York, USA; 6 Amine Pharma Research Institute, Innovation Plaza at Chiba University, Chiba, Japan

**Keywords:** glycocalyx, heparan sulfate, chondroitin sulfate, heparanase, acrolein, ischemic stroke, stroke, hyaluronan, hyaluronidase

## Abstract

Infiltration of peripheral immune cells after blood-brain barrier dysfunction causes severe inflammation after a stroke. Although the endothelial glycocalyx, a network of membrane-bound glycoproteins and proteoglycans that covers the lumen of endothelial cells, functions as a barrier to circulating cells, the relationship between stroke severity and glycocalyx dysfunction remains unclear. In this study, glycosaminoglycans, a component of the endothelial glycocalyx, were studied in the context of ischemic stroke using a photochemically induced thrombosis mouse model. Decreased levels of heparan sulfate and chondroitin sulfate and increased activity of hyaluronidase 1 and heparanase (HPSE) were observed in ischemic brain tissues. HPSE expression in cerebral vessels increased after stroke onset and infarct volume greatly decreased after co-administration of *N*-acetylcysteine + glycosaminoglycan oligosaccharides as compared with *N*-acetylcysteine administration alone. These results suggest that the endothelial glycocalyx was injured after the onset of stroke. Interestingly, scission activity of proHPSE produced by immortalized endothelial cells and HEK293 cells transfected with hHPSE1 cDNA were activated by acrolein (ACR) exposure. We identified the ACR-modified amino acid residues of proHPSE using nano LC–MS/MS, suggesting that ACR modification of Lys^139^ (6-kDa linker), Lys^107^, and Lys^161^, located in the immediate vicinity of the 6-kDa linker, at least in part is attributed to the activation of proHPSE. Because proHPSE, but not HPSE, localizes outside cells by binding with heparan sulfate proteoglycans, ACR-modified proHPSE represents a promising target to protect the endothelial glycocalyx.

The breakdown of the blood-brain barrier (BBB) and the infiltration of monocytes and neutrophils are critical steps toward severe inflammation of acute ischemic stroke (1, 2). Post-ischemic inflammation is elicited by the damage-associated molecular patterns, especially high mobility group box 1 (HMGB1) and peroxiredoxin, of necrotic brain tissues (2). HMGB1 released extracellularly several hours (hyperacute phase) after stroke onset promotes BBB disruption (3), whereas macrophage activation by peroxiredoxin occurs in the acute and subacute phases (12–72 h after onset) (4). Induction of matrix metalloprotease (MMP), especially MMP9 in the acute and subacute phases, is also involved in the disruption of tight-junctions of cerebral vessels (5). Thus, the effective protection of the BBB after the onset of ischemic stroke is important in attenuating post-ischemic inflammation.

The glycocalyx, an extracellular layer of endothelial cells (6), is composed of proteoglycans and sulfated glycosaminoglycans (GAGs), including chondroitin sulfate (CS) and heparan sulfate (HS). Hyaluronan (HA), nonsulfated glycosaminoglycans that exist as a free form, is also a major component of the endothelial glycocalyx (7).

The glycocalyx functions as a barrier to circulating cells; however, enzymatic degradation, especially HS degradation by heparanase (HPSE) (8, 9), an endo-β-glucuronidase, induces inflammatory responses in conditions such as sepsis (10), proteinuria (11), and diabetic nephropathy (12). Although increased plasma levels of GAGs and proteoglycans were reported in patients after ischemic stroke onset (13), the relationship between stroke severity and glycocalyx dysfunction is not understood.

HPSE is synthesized via its precursor pre-proHPSE (68 kDa), and the signal peptide of pre-proHPSE is cleaved upon entry into the endoplasmic reticulum (8, 9). The resulting 65-kDa proHPSE is transported to the Golgi apparatus and is secreted extracellularly. Thereafter, proHPSE, which binds to the HS proteoglycan on cellular membranes, is localized within late endosomes and lysosomes, and the 6-kDa linker (residues 110–157) is subsequently removed by cathepsin L to synthesize the active form of proHPSE. Processed and activated HPSE is estimated to be 100-fold more active than proHPSE (9). Thus, HPSE plays homeostatic roles in regulating HS turnover as a lysosomal protein, whose activity is pH-dependent (pH 5.5). The activation of HPSE by the secreted cathepsin L or the localization of HPSE on macrophage surface contributes to the disruption of extracellular matrices, which are involved in inflammation including diabetes, Alzheimer's disease, and cancer metastasis (8, 9). However, the detailed mechanism of extracellular degradation of HS by HPSE is not fully understood.

In the present study, we found that degradation of HS and CS in infarct lesions occurred during the hyperacute phase in ischemic stroke mice. Increased hyaluronidase (HYAL) 1 levels and HPSE activity in infarct lesions, and induction of HPSE level in cerebral vessels after stroke onset, were observed. In addition, reduction of infarct volume by intraperitoneal co-administration of enoxaparin (heparin oligosaccharides as an HPSE inhibitor) and CS oligosaccharides (as an HYAL1 inhibitor) with *N*-acetylcysteine (NAC) was greater than that observed with a single dose of NAC. Interestingly, HS degradation and induction of proHPSE expression, via exposure to acrolein (ACR: CH_2_ = CH − CHO), a highly reactive unsaturated aldehyde produced during the stroke (14), were observed in immortalized human brain microvascular endothelial cells (HBMEC/ciβ) (15). Findings of nano LC–MS analysis suggest that ACR modification of Lys^139^ (6-kDa linker), Lys^107^, and Lys^161^ (located in the immediate vicinity of the 6-kDa linker) is partly attributable to the activation of proHPSE. These results suggest that proHPSE is a promising target to protect BBB functions after ischemic stroke onset.

## Results

### Degradation of HS and CS by HPSE and HYAL1 in infarct lesions of mice

**Figure 1:**
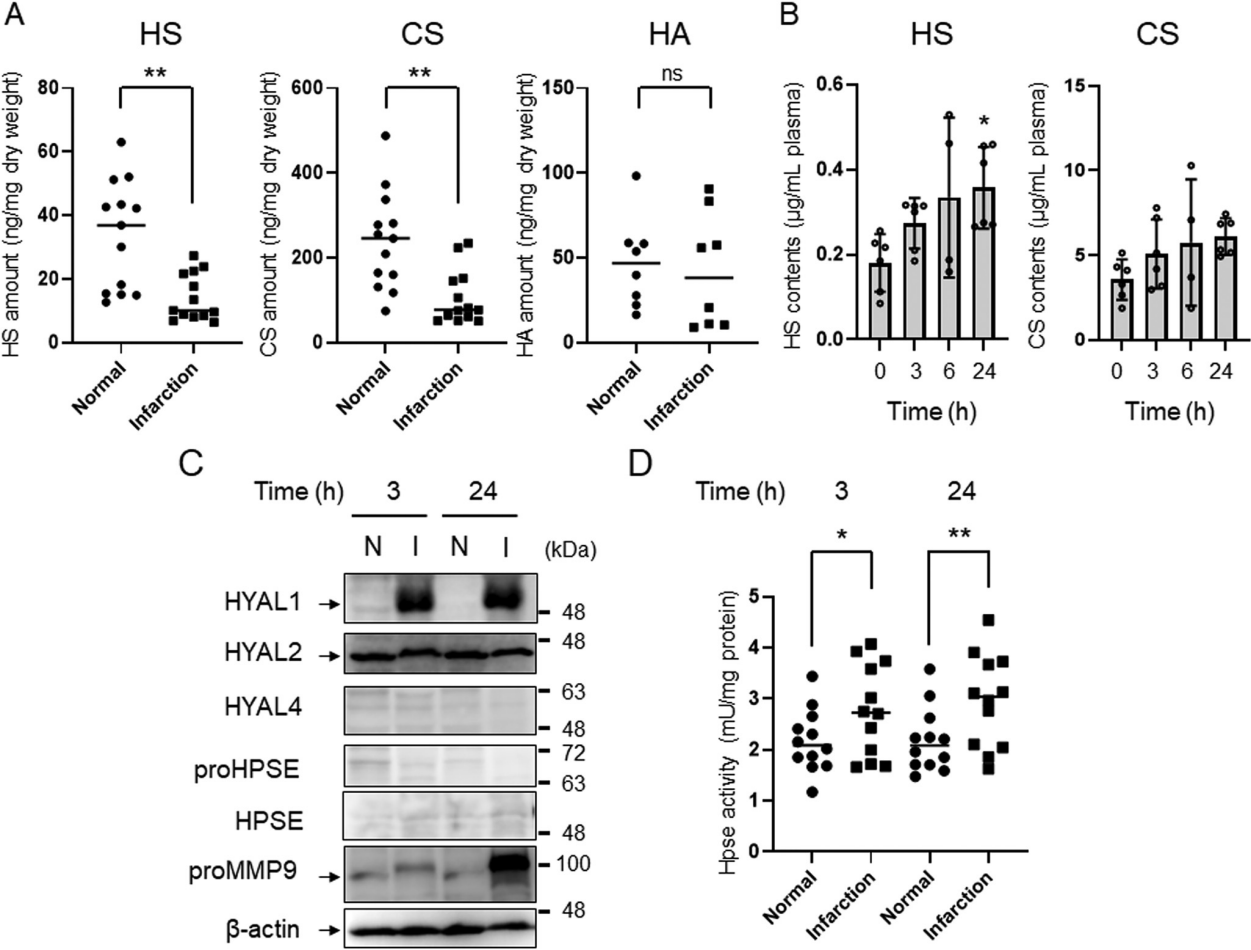
**Degradation of glycosaminoglycans after stroke induction.***A*, levels of HS, CS, and HA in infarct region or normal area (in the contralateral hemisphere) of mouse brain tissues at 24 h after stroke induction. Extraction of GAGs and analysis of their unsaturated disaccharides were performed as described under “Experimental procedures.” Compositions of unsaturated disaccharides of HS and CS are shown in Fig. S1. *B*, plasma contents of HS and CS after onset of ischemic stroke. Data are expressed as the mean ± S.D. (*error bars*). *C*, expression levels of HYAL, HPSE, and MMP9 in infarct region or normal area of mouse brain tissues after onset of ischemic stroke. Western blotting of each protein was performed using 20 µg of protein extracted from tissues. Experiments were repeated three times and the same results were obtained. *N*, normal, *I*, infarct region. *D*, HPSE activity in the infarct region or normal area after onset of stroke. HPSE activity in tissue extracts (150 µg of protein) was measured using a heparan-degrading enzyme assay kit. *Horizontal line* indicates median. *p < 0.05; **p < 0.01; *ns*, not significant.

First, expression levels of glycosaminoglycans, including HS, CS, and HA, in infarct lesions or normal tissues in mice were investigated by unsaturated disaccharide analysis using HPLC. As shown in Fig. 1*A*, levels of HS and CS in infarct lesions were decreased compared with those in normal brain tissues. The disaccharide compositions of HS and CS in normal brain tissue and infarct lesions were almost identical (Fig. S1). No change in HA level was observed (Fig. 1*A*). Plasma HS and CS concentrations increased from 3 h after stroke onset and continued to increase up to 24 h (Fig. 1*B*). These results suggest that degradation of HS and CS in brain tissues occurs during hyperacute and acute phases after stroke onset. Glycocalyx dysfunction, due to HPSE or MMP9, in several diseases (10, 11, 12, 16) and increased levels of HYAL1 and 2 in infarct lesions of ischemic stroke patients were reported (17). Additionally, HYAL1 expression levels were upregulated in microvessels and neurons, whereas nuclear translocation of HYAL2 was stimulated in neurons of peri-infarct regions (17). In the HYAL family, HYAL1 and 2 exhibit HA-hydrolyzing activity, whereas HYAL1 and 4 exert CS-hydrolyzing activity (18). Thus, expression levels of HPSE, MMP9, and HYAL1-4 in infarct lesions at 3 and 24 h after stroke onset were investigated using Western blotting. As a result, expression levels of HYAL1, but not HYAL2, increased at 3 and 24 h post-stroke onset, whereas HYAL4 and HPSE were not detected (Fig. 1*C*). Because HS level decreased in infarct lesions, scission activity of HPSE was measured, and it was found that HPSE activity was elevated 3 and 24 h after the stroke induction (Fig. 1*D*). Although MMP9 expression was low at 3 h, marked upregulation was observed at 24 h (Fig. 1*C*). Taken together, these results suggest that disruption of GAGs and induction of HYAL1 and HPSE in infarct lesions occurred at the hyperacute phase.

**Figure 2:**
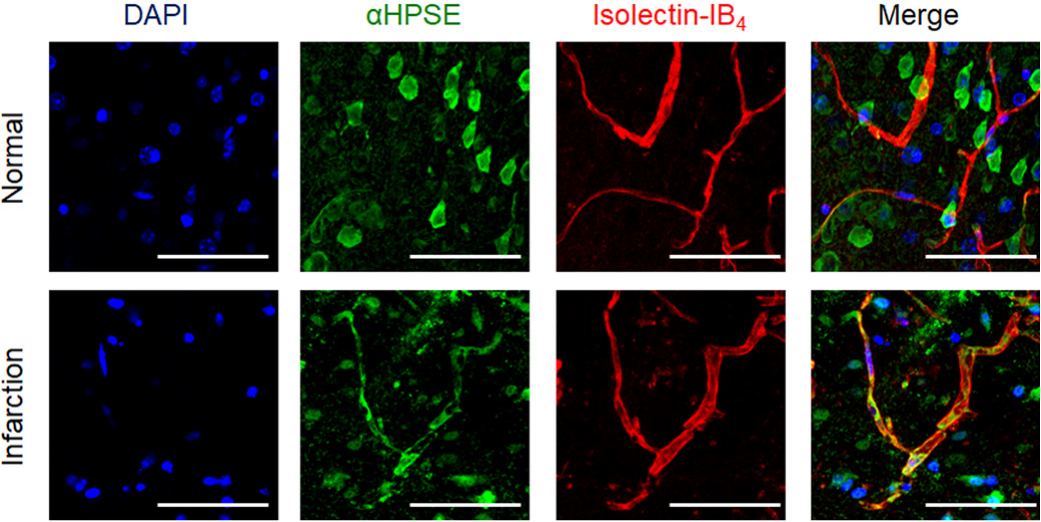
**Induction of HPSE expression in microvascular endothelial cells 24 h after stroke onset.** Mouse brain was removed 24 h after the onset of ischemic stroke. The nuclei, HPSE, and endothelial cells were stained using DAPI, anti-HPSE antibody (H-80), and isolectin-B4, respectively (Table S2). *Bar* = 50 µm. Note that the primary HPSE (H-80) antibody can recognize 101–180 amino acids of HPSE and proHPSE. Experiments were repeated three times, and reproducible results were obtained.

**Figure 3:**
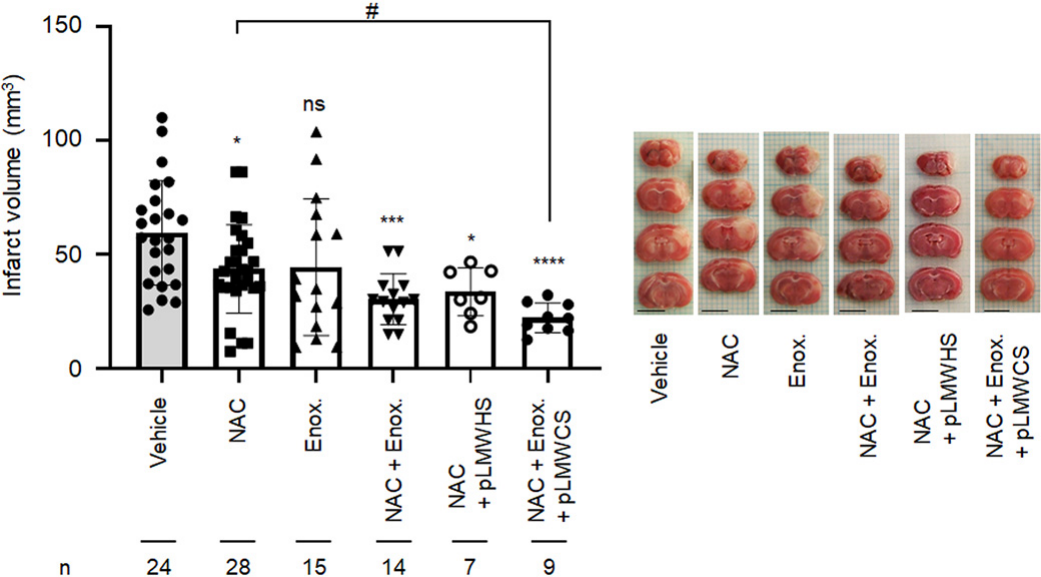
**Effect of coadministration of NAC, LMWH, and pLMWCS on the size of infarct volume.** Preparation of PIT mouse model, triphenyltetrazolium chloride staining of brain tissue, and analysis of infarct volume were carried out as described in “Experimental procedures.” Intraperitoneal administration of NAC (250 mg/kg), enoxaparin (2.5 mg/kg), pLMWHS (100 mg/kg), and pLMWCS (100 mg/kg) in PBS was performed immediately after induction of infarction. The therapeutic range (0.3–0.7 IU/ml) for anti-FXa activity was observed at 0.5–4 h after injection of enoxaparin (2.5 mg/kg) (data not shown). Data are expressed as the means ± S.D. (*error bars*). *p < 0.05, ***p < 0.001, ****p < 0.0001 against vehicle. #*p* < 0.05 against NAC. *ns*, not significant. *Bar* = 5 mm. *Enox.*, enoxaparin.

Upregulation of HPSE in vascular cells, neurons, and astrocytes of mouse brain was reported 7 days post-ischemia (19). Because HPSE protein levels increased in vascular cells 24 h post stroke induction (Fig. 2), the impact of glycocalyx protection on the infarct volume was next examined using low-molecular-weight heparin (LMWH: enoxaparin) as an inhibitor of HPSE (20) and low-molecular-weight CS (pLMWCS) as an HYAL1 inhibitor. NAC, a precursor of GSH, which exerts antioxidant activity (14), reduced infarct volume by 35% in mice (Fig. 3). When LMWH (enoxaparin) and pLMWCS were intraperitoneally administered to mice in combination with NAC, reduction of infarct volume was in the order of administration, namely NAC+enoxaparin+pLMWCS > NAC+enoxaparin > NAC > enoxaparin=none (Fig. 3). To exclude the possibility that the anticoagulant activity of enoxaparin disrupted thrombosis formation, a nonanticoagulant low-molecular-weight heparan sulfate (pLMWHS) prepared by photolysis (21) was used instead of enoxaparin. As a result, a reduction of infarct volume was observed in mice that received NAC+pLMWHS which was greater than the reduction observed in mice receiving NAC alone. These results suggest that glycocalyx impairment aggravates inflammation after stroke onset.

### Induction of proHPSE expression and its activity in brain microvascular endothelial cells due to ACR exposure

**Figure 4:**
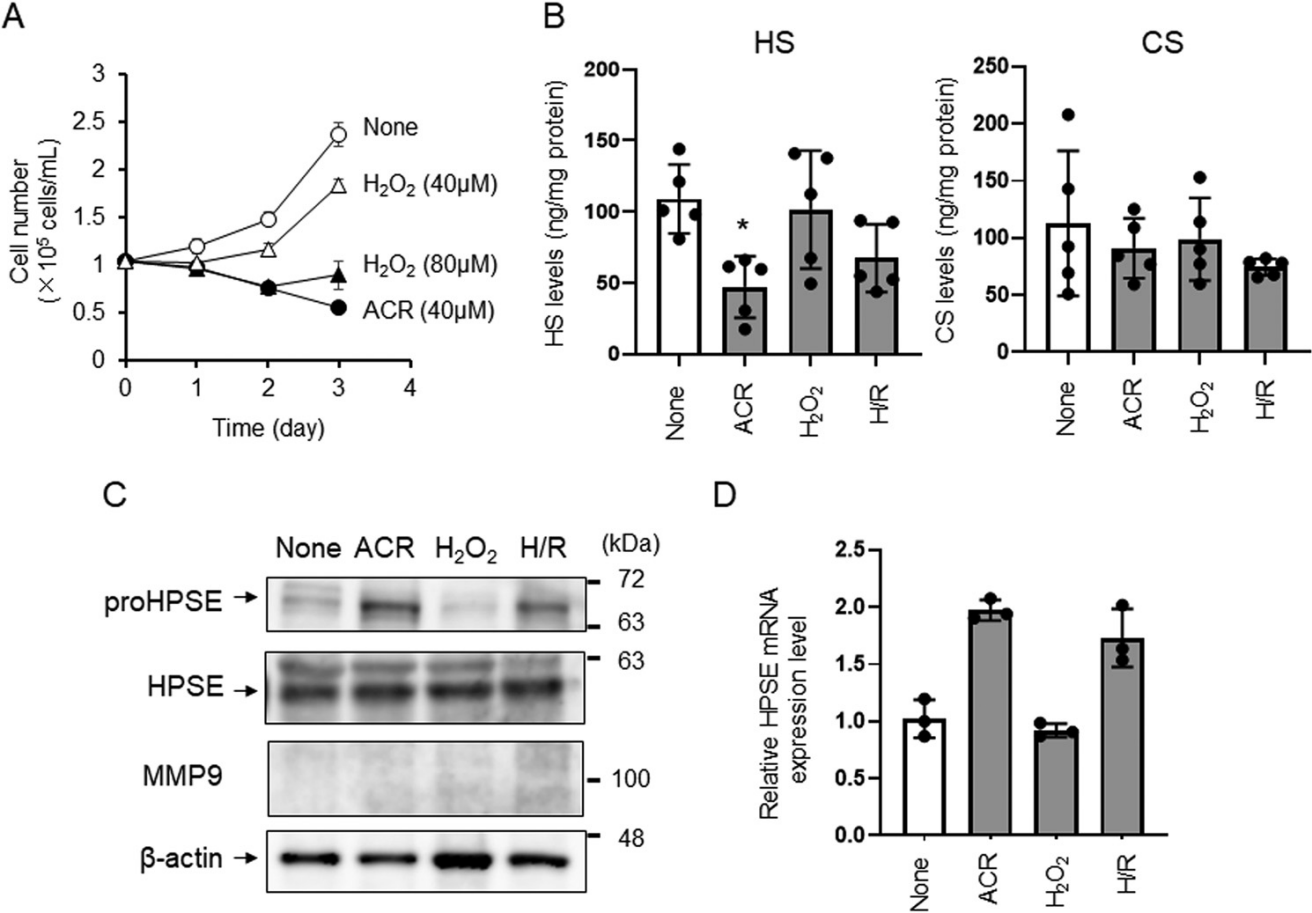
**Acrolein induces HS degradation in HBMEC/ciβ.** Cell growth (*A*), expression levels of HS and CS (*B*), expression levels of HPSE, proHPSE, and MMP9 (*C*), and expression level of *HPSE* mRNA (*D*) of HBMEC/ciβ exposed to acrolein (ACR) or hydrogen peroxide (H_2_O_2_). *A*, to examine the toxicity of ACR and H_2_O_2_, 1.0 × 10^5^ cells of HBMEC/ciβ were inoculated into 35-mm dishes and cultured for 72 h. *B*, GAGs were extracted using 1.0 × 10^7^ cells of HBMEC/ciβ and analyzed using HPLC. *C*, Western blotting of each protein was performed using 20 µg of protein extracted from the cell lysate. Experiments were repeated three times and the same results were obtained. *D*, data were calculated using the 2^−ΔΔCt^ method. Transcription of the housekeeping gene *GAPDH* was used to normalize data. *A*, *B*, and *D*, data are expressed as the mean ± S.D. (*error bars*). *p < 0.05 against none.

Several reports indicate that oxidative stress is induced in cerebral ischemia through the production of reactive oxygen species and exposure to ACR (1, 14). ACR, produced by polyamine oxidation (22), lipid oxidation (23), and myeloperoxidase in neutrophils (24), can attack amino acid residues in target proteins, resulting in *N*^ε^-(3-formyl-3,4-dehydropiperidino)lysine and *N*^ε^-(3-methylpyridinium)lysine, which induce structural changes to proteins, thereby altering activity and function (22, 25). Thus, we examined the effects of exposure to hydrogen peroxide (H_2_O_2_) or ACR on expression levels of GAGs in HBMEC/ciβ (15). Complete inhibition of cell growth was accomplished upon extracellular addition of 40 μm of ACR or 80 μm of H_2_O_2_ post-culture for 72 h (Fig. 4*A*). Levels of HS, but not CS, appeared decreased in endothelial cells exposed to ACR but not H_2_O_2_ (Fig. 4*B*). The expression levels of proHPSE (inactive), but not HPSE (active), increased in response to ACR exposure (Fig. 4*C*), despite the ACR-induced increase of *HPSE* mRNA (Fig. 4*D*). Although HPSE transcription is normally silenced (except for cells such as endothelial cells, placenta, leukocytes, and platelets) by tumor suppressor p53 (9, 26), it was reported that hypoxia induces HPSE expression through activation of NF-κB (27). Under our culture conditions, HS degradation and induction of *HPSE* mRNA in HBMEC/ciβ was also facilitated by hypoxia/reperfusion (Fig. 4, *C* and *D*). Although HPSE transcription may be induced by ACR through inhibition of p53 DNA binding activity (28) rather than activation of NF-κB (24), the mechanism by which ACR induces HPSE activity remains to be elucidated.

### Scission activity of proHPSE was activated by ACR exposure

**Figure 5:**
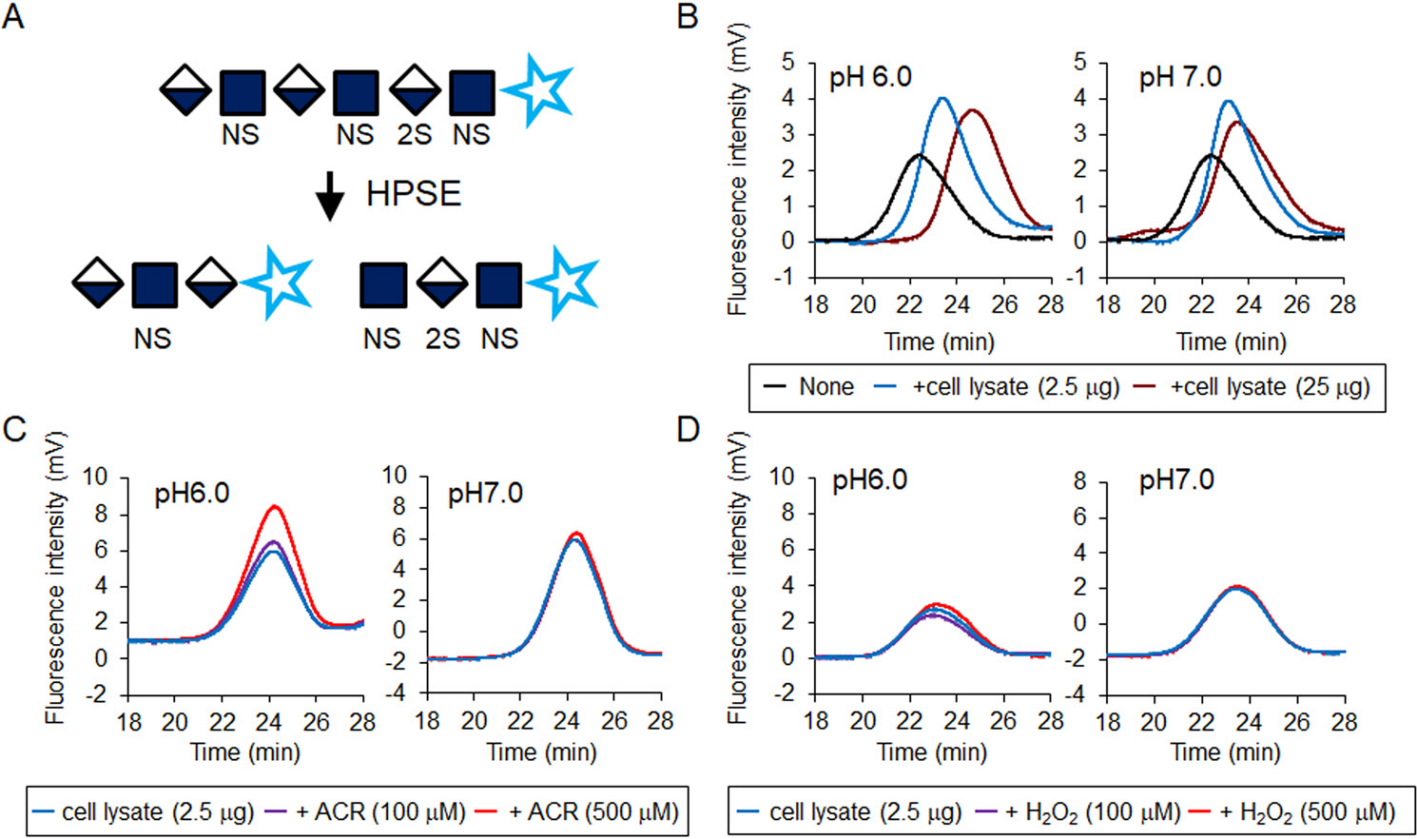
**Effect of acrolein or hydrogen peroxide on HPSE activity of HEK293 cells transfected with *hHPSE* cDNA.***A*, HPSE activity can be measured using GPC with post-column derivatization. Aldehyde groups at the reducing end of polysaccharides react with 2-cyanoacetoamide as a fluorogenic post-labeling reagent. In general, scission activity (HPSE activity) is evaluated by GPC through the monitoring of the prelabeled heparin degradation or by a heparan-degrading enzyme assay kit with prelabeled heparin (Fig. S2A). However, it was difficult to prepare the free form of proHPSE from conditioned medium, and measurement of scission activity of proHPSE by heparan-degrading enzyme assay kit was also disturbed by unfractionated heparin (data not shown). Therefore, unfractionated heparin which is supplemented to conditioned medium was directly used as a substrate of proHPSE, and post-label derivatization was employed to evaluate the scission activity. *B*, HEK293 cells transfected with *hHPSE1* cDNA was lysed in lysis buffer (pH 6.0 or pH 7.0), and the resulting cell lysates (2.5 or 25 µg) were incubated with unfractionated heparin (2.0 µg). Extraction and GPC were performed as described under “Experimental procedures.” *C*, effect of ACR on HPSE activity. *D*, effect of H_2_O_2_ on HPSE activity. Experiments were repeated in triplicate with reproducible results.

**Figure 6:**
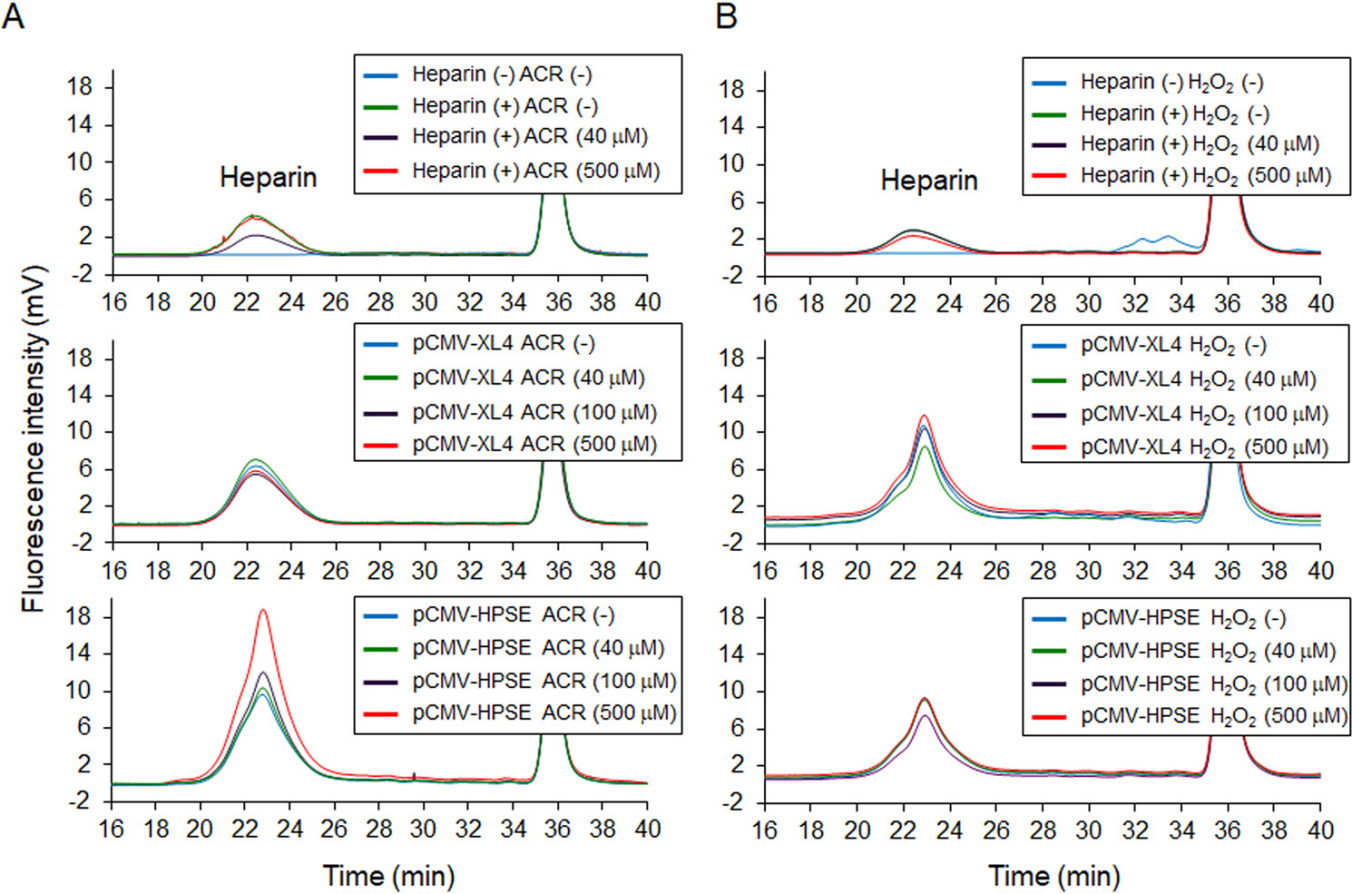
**Activated scission activity of proHPSE by acrolein exposure.** Effect of ACR (*A*) or H_2_O_2_ (*B*) on the scission activity of proHPSE in conditioned medium containing 50 µg of unfractionated heparin. Preparation of conditioned medium of HEK293 cells transfected with *hHPSE1* cDNA and measurement of scission activity of proHPSE by GPC were carried out as described under “Experimental procedures.” Experiments were repeated in triplicate with reproducible results.

HEK293 cells transfected with *hHPSE1* cDNA were exposed to 40 μm ACR for 48 h to clarify the activation of HPSE activity by ACR. As a result, HPSE activity in HEK293 cells appeared slightly increased because of ACR exposure (Fig. S2A). HEK293 cells transfected with *hHPSE1* cDNA produced not only HPSE but also proHPSE, and it seems that cathepsin L–dependent (29) maturation of HPSE was not influenced by ACR exposure (Fig. S2B). Thus, the effect of ACR on the activity of HPSE was examined using unfractionated heparin as a substrate. Because aldehyde groups at the reducing end of heparin react with 2-cyanoacetoamide as a fluorogenic post-labeling reagent, HPSE activity can be measured using gel permeation chromatography (GPC) with post-column derivatization (Fig. 5*A*). Thus, increased fluorescence intensity and elution time of heparin reflect the enzymatic activity of HPSE. HEK293 cells transfected with *hHPSE1* cDNA were lysed in a buffer solution adjusted to pH 6.0 or pH 7.0, and the resulting cell lysates were incubated with 2 μg of unfractionated heparin. After incubation at 37 °C for 24 h, resulting degraded heparin was analyzed using GPC with fluorescence detection. As a result, HPSE activity depended on cell lysate proteins, and activity at pH 6.0 appeared greater than that at pH 7.0 (Fig. 5*B*). Because pH dependence of HPSE activity (30) was suggested by GPC, the effect of ACR or H_2_O_2_ on HPSE activity was next examined. As a result, HPSE activity was activated by ACR at pH 6.0 but not at pH 7.0 (Fig. 5*C*). In contrast, HPSE activity was not affected by H_2_O_2_ exposure (Fig. 5*D*). This result suggests that the modification of Lys residues of HPSE protein by ACR stimulates scission activity. However, cell lysates from HEK293 cells transfected with *hHPSE1* cDNA contain both HPSE (active) and proHPSE (inactive) proteins (see Fig. S2B), and their localization in cells differs slightly. ProHPSE exists on the plasma membrane by binding with the HS moiety of proteoglycans, and HPSE functions as a lysosomal protein after endocytic uptake of HPSE and HS proteoglycan complexes (31). ProHPSE can be obtained from the conditioned medium of HPSE producing cells supplemented with unfractionated heparin (31) (Fig. S3). Thus, the effect of ACR or H_2_O_2_ on the enzymatic activity of proHPSE was examined by GPC. Results indicated that scission activity of proHPSE was markedly activated in an ACR concentration-dependent manner (Fig. 6). Maturation of proHPSE to HPSE due to ACR exposure in conditioned medium was not observed (Fig. S3). This result suggests that ACR can activate the enzymatic activity of proHPSE.

### Identification of ACR-conjugated amino acid residues in proHPSE

**Figure 7:**
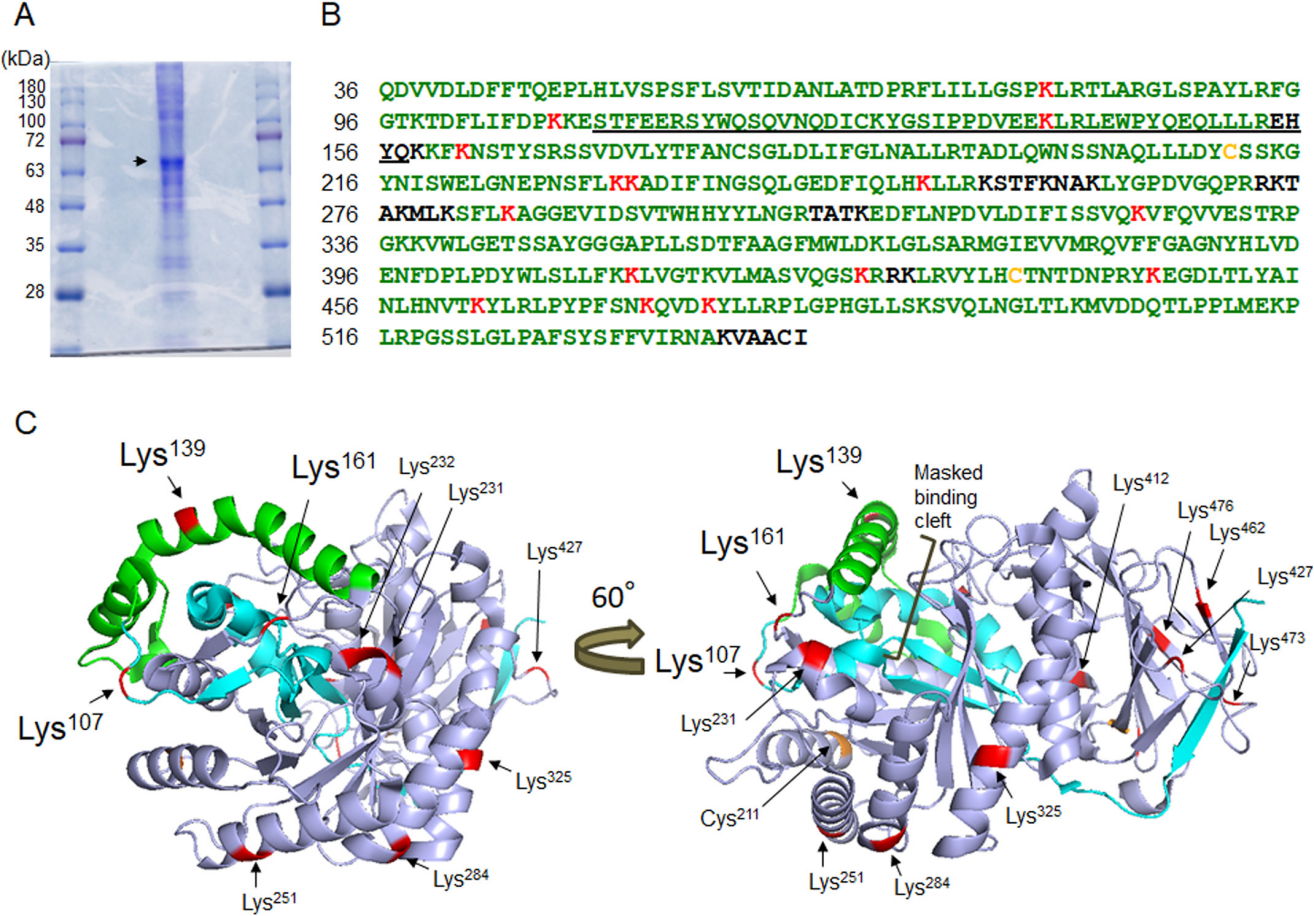
**Identification of acrolein-modified amino acid residues in proHPSE.***A*, SDS-PAGE of proHPSE-rich fraction from conditioned medium of HEK293 cells transfected with *hHPSE1* cDNA. The 65-kDa protein was recognized by Coomassie Brilliant Blue staining. *B*, amino acid sequences of proHPSE were determined using nano LC–MS/MS. Identified amino acid sequences are shown in *green*, based on the results of peptide analysis by nano LC–MS/MS. The acrolein-modified amino acids of proHPSE are shown in *yellow* (incubated without acrolein for 24 h) or *red* (exposed to 500 µm acrolein for 24 h). The 6-kDa linker is *underlined*. *C*, location of acrolein-modified amino acid residues on proHPSE (PDB ID: 5LA4). *Cyan*, 8-kDa subunit; *green*, 6-kDa linker; *light blue*, 50-kDa subunit.

Peptides derived from the proHPSE-rich fraction (Fig. 7*A*), treated with or without 500 μm ACR at 37 °C for 24 h, were analyzed using nano LC–MS/MS. Peptides containing ACR-modified residues were explored because cysteine (Cys), histidine (His), and lysine (Lys) in protein are nucleophilic targets of ACR (23, 32). Mass spectra of analyzed peptides containing ACR-modified residues and peptide sequences in proHPSE are shown in Fig. S4, Table S1, and Fig. 7*B*. Interestingly, proHPSE produced by HEK293 cells without ACR exposure was already modified at Cys^211^ and Cys^437^ in the 50-kDa subunit (Fig. 7*B*). When proHPSE was incubated with 500 μm ACR, 15 Lys residues were further modified, namely Lys^79^ and Lys^107^ in the 8-kDa subunit, Lys^139^ in the 6-kDa linker (residues 110–157), and lysines 161, 231, 232, 251, 284, 325, 412, 427, 446, 462, 473, and 477 in the 50-kDa subunit (Fig. 7*B*). The positions of ACR-modified Lys and Cys residues do not fully overlap with the HPSE binding cleft of Lys residues (Lys^158^, Lys^159^, Lys^161^, and Lys^231^) (33) or a disulfide bond between Cys^437^ and Cys^542^, which are required for activation (34). In addition, ACR-modified Lys residues are not close to the amino acids (Asp^62^, Asn^64^, Thr^97^, Lys^159^, Asn^224^-Glu^225^, Gln^270^, Arg^272^, Arg^303^, Glu^343^, Gly^349^-Gly^350^, and Gly^389^) involved in binding the HS substrate (33). The 3D crystal structure of proHPSE indicates that a large helical domain of the 6-kDa linker (shown in *green*) lies directly above the active site cleft, inhibiting binding of the bulky HS substrates to HPSE (Fig. 7*C*) (35). Lys^107^ and Lys^161^ in both end hinge regions, and Lys^139^ in the 6-kDa linker, are further modified by ACR exposure (Fig. 7*C*). The final loop of the linker (His^155^-Lys^159^) is more flexible than the rest of the proHPSE linker (35). The disorder of this loop containing Lys^156^ is thought to be important for recognition by cathepsin L to induce maturation of HPSE protein without structural alterations (29, 35). Given that dynamic structural changes of protein-disulfide isomerase by ACR influence enzymatic activity (25), ACR modification of Lys^107^ and Lys^161^ in both end hinge regions and Lys^139^ in the 6-kDa linker may be critical to generate scission activity of proHPSE by disrupting preexisting secondary structures of the 6-kDa linker.

## Discussion

We discovered that HS in infarct lesion was degraded by HPSE after onset of stroke (Fig. 1). Expression level of proHPSE increased in the vascular cells in ischemic stroke mouse model (Fig. 2) and HBMEC/ciβ exposed to ACR or hypoxia/reperfusion (Fig. 4). Scission activity of proHPSE, derived from the conditioned medium of HEK293 cells supplemented with heparin, was generated by ACR exposure (Fig. 6). Failure of substantial activation of HPSE (active form) by ACR exposure was also confirmed using recombinant HPSE (R&D Systems) and a heparan-degrading enzyme assay kit (TaKaRa Bio) containing prelabeled heparin (data not shown). Based on these observations, we concluded that proHPSE, activated by ACR, together with HPSE, secreted from immune cells, accelerate the degradation of the endothelial glycocalyx after stroke onset. Indeed, coadministration of enoxaparin and NAC is more effective than a single dose of NAC or enoxaparin (Fig. 3). In this experiment, depolymerized oligosaccharides of heparin, HS, and CS were employed because administration of larger GAG molecules was generally unusable in terms of bioavailability, plasma *t*_1/2_, and side effects, especially bleeding by heparin overdose (36). Therefore, HPSE inhibitor, especially anti-heparanase mAbs (8) currently developed for cancer, might exert more protective effects on the glycocalyx during the acute phase of ischemic stroke.

We observed CS degradation and upregulation of HYAL1 expression in infarct lesions after onset of stroke (Fig. 1). Although no significant HA decrease was observed after the stroke induction (Fig. 1), induced HYAL1, at least in part, may degrade HA after stroke onset. Induction of tumor necrosis-stimulated gene 6 (TSG-6) in the peri-infarct region was observed in ischemic stroke patients (17), as was the irreversible complex formation between the heavy chain of bikunin and HA oligosaccharide (37) mediated by TSG-6 promotes leukocyte adhesion (38). Curiously, ACR-induced CS degradation was not observed in HBMEC/ciβ (Fig. 4*B*) and other cell types (data not shown), despite the fact that weak expression of HYAL1 was observed in blood vessels and neurons of infarct lesion patients (17). Given that upregulation of HYAL1, but not HYAL2, was increased in macrophages treated with receptor activator of NF-κB ligand (39), accumulation and activation of macrophages may contribute to CS degradation in infarct lesions.

CS, keratan sulfate, and HS have been reported to regulate neuronal plasticity and axon guidance in the extracellular matrix of the central nervous system (40). CS and keratan sulfate are the major inhibitors of axonal growth, whereas HS promotes its growth by interacting with the neuronal receptors PTPRσ and LAR (40, 41, 42). These effects on axonal growth depend on the length of the sulfated disaccharide sequence of the proteoglycans and their position in the glycan chain (40). In the chronic phase after central nervous system injury, accumulated CS proteoglycans in glial scar hinder axonal regeneration (43, 44). However, the role of CS (and HS) degradation in the acute phase (in this study) of glial scar formation remains unclear. Studies investigating the role of CS degradation on glial scar formation are underway.

## Experimental procedures

### Photochemically induced thrombosis model mice

All animal experiments were approved by the Institutional Animal Care and Use Committee of Chiba University and carried out according to the Guidelines for Animal Research of Chiba University. Photochemically induced thrombosis (PIT) model mice were prepared as described previously (14). The thrombotic occlusion of the middle cerebral artery in male C57B/L mice (8-week-old) weighing 22–26 g was performed using photoillumination (wavelength 540 nm; L4887, Hamamatsu Photonics, Hamamatsu, Japan) after intravenous administration of Rose Bengal (20 mg/kg). After induction of stroke, 2-mm-thick coronal brain slices were prepared and incubated with 5% triphenyltetrazolium chloride solution at 37 °C for 30 min. Infarct volume was analyzed using the National Institutes of Health image program. Oligosaccharides, that is pLMWHS (molecular weight 5000) and pLMWCS (molecular weight 5000), were prepared as described previously (21, 45). Enoxaparin (Sanofi-Aventis, Cambridge, MA) was desalted using Amicon Ultra Centrifugal Filter 30K device (EMD Millipore, Burlington, MA) and lyophilized. Intraperitoneal administration of NAC (14) (250 mg/kg), enoxaparin (2.5 mg/kg), pLMWHS (100 mg/kg), and pLMWCS (100 mg/kg) in PBS was performed immediately after induction of infarction.

### HPLC of unsaturated disaccharides of HS, CS, and HA

Brain tissues (5–20 mg wet weight) and plasma (0.24–0.45 ml) were homogenized with four volumes of acetone overnight to remove lipids and dried completely. Resulting dried tissue samples or cultured cells (1 × 10^7^) (without acetone treatment) were treated with actinase E (0.25 mg/ml) in 400 μl of 50 mm Tris acetate buffer (pH 8.0) at 45 °C for 3 days. Microscale isolation of GAGs by Vivapure Q mini H spin column (Sartorius Stedim Biotech GmbH, Göttingen, Germany) centrifugation was performed as described previously (46, 47). The crude GAGs were incubated in the reaction mixture (35 μl) containing 28.6 mm Tris acetate (pH 8.0) and 50 milliunits of Chondroitinase ABC (Seikagaku Corp., Tokyo, Japan). To analyze HA contents in brain tissues, 50 milliunits of Chondroitinase ACII (Seikagaku Corp., Tokyo, Japan) was also added to the reaction mixture. After 16 h at 37 °C, depolymerized GAG samples were boiled and evaporated, and unsaturated disaccharides from CS and HA were collected using the Amicon Ultra Centrifugal Filter 30K device. The remaining HS samples in filters of spin columns were transferred to new microtubes and incubated in 16 μl of reaction mixture (pH 7.0) containing 1 milliunit of heparinase I (Seikagaku Corp.), 1 milliunit of heparinase II (Iduron, Manchester, UK), 1 milliunit of heparinase III (Seikagaku Corp.), 31.3 mm sodium acetate, and 3.13 mm calcium acetate for 16 h at 37 °C. Unsaturated disaccharide analysis, using reversed-phase ion-pair chromatography with sensitive and specific post-column detection, and HPLC analysis for polyamines were performed as described previously (46). Expression levels of HS or CS were expressed as total amounts of unsaturated disaccharides. Protein contents were determined using the method developed by Lowry *et al.* (48).

### Cell culture

HBMEC/ciβ was cultured as described previously (15). Briefly, HBMEC/ciβ (1.7 × 10^6^ cells) was cultured in VascuLife Basal Medium (Lifeline Cell Technology, Frederick, MD) supplemented with 10% FBS, 2 mm l-Alanyl-l-Glutamine Solution (FUJIFILM Wako Pure Chemical Co., Tokyo, Japan), 4 μg/ml blasticidin S (InvivoGen, San Diego, CA), 1% penicillin, and 1% streptomycin in an atmosphere of 5% CO_2_/95% air at 33 °C. HBMEC/ciβ (1.0 × 10^7^ cells) were exposed to 24 h of hypoxia (pO_2_ < 1%) using the BIONIX-1 hypoxic cell culture kit (Sugiyamagen, Tokyo, Japan) and 30-min reoxygeneration to examine the effect of hypoxia/reperfusion on the expression level of GAGs (49). HEK293 cells were cultured in DMEM supplemented with 10% FBS, 100 units/ml penicillin G, and 50 units/ml streptomycin in an atmosphere of 5% CO_2_/95% air at 37 °C.

### Western blotting analysis

Western blotting analysis was performed using the method developed by Nielsen *et al.* (50) using Amersham^TM^ ECL Select^TM^ Western blotting reagents (GE Healthcare Life Sciences, Buckinghamshire, UK). Antibodies used in this study are listed in Table S2. Protein levels were quantified using an ImageQuant^TM^ LAS 4000 (GE Healthcare Life Sciences). Protein contents were determined using the Lowry *et al.* method (48).

### Immunohistochemical staining

PIT model mice, at 24 h after stroke onset, were perfused intracardially with PBS followed by 4% paraformaldehyde (PFA) in PBS. The entire brain was removed and immersed in 800 μl of 4% PFA at 4 °C for 24 h. After dehydration in order using 10%, 20%, and 30% sucrose in 0.1 m PBS, brain tissues were embedded into O.C.T. compound (Sakura FineTek, Tokyo, Japan) and frozen at −80 °C. Thereafter, 25-μm-thin sections were prepared using Cryostat (CM3050S; Leica, Wetzlar Germany). The tissue section was fixed again with 4% PFA, washed with PBS, and immersed in Histo VT one (Nacalai Tesque, Tokyo, Japan) at 70 °C for 20 min, followed by incubation with 0.2% Triton X-100 in PBS at room temperature for 10 min. After washing with 0.5% Tween 20 in PBS, tissue sections were blocked with 3% BSA and 0.5% Tween 20 in PBS. Primary antibodies of HPSE (H-80) and secondary antibodies used in this study are shown in Table S2. Tissue sections were observed using a confocal microscope LSM 780 (Carl Zeiss, Oberkochen, Germany).

### Measurement of mRNA

Total RNA was isolated using the RNeasy Mini Kit (Qiagen GmbH, Hilden, Germany). DNase treatment of RNA samples prior to reverse transcription was performed using RQ1 RNase-Free DNase (Promega). After removal of degraded DNA using the Amicon Ultra-0.5 ml Centrifugal Filters 3K device, synthesis of the first-strand cDNA from total RNA was performed using SuperScript^TM^ II Reverse Transcriptase (Thermo Fisher Scientific) according to the manufacturer's instructions. Quantitative PCR, with SYBR Advantage qPCR Premix (TaKaRa Bio), was performed using primers 5′-CTCGAAGAAAGACGGCTA-3′ and 5′-GTAGCAGTCCGTCCATTC-3′ to measure the transcriptional level of the *hHPSE* gene in HBMEC/ciβ. Transcription of the housekeeping gene *GAPDH* was used to normalize data. Primers used to amplify the *hGAPDH* gene are 5′-AGCCACATCGCTCAGACAC-3′ and 5′-GCCCAATACGACCAAATCC-3′, respectively. The transcript levels were calculated using the 2^−ΔΔCt^ method.

### Heparanase activity

HEK293 cells were transfected with pCMV-*hHPSE1* (OriGene Technologies, Inc., Rockville, MD) as follows. Plasmid solution containing 1.5 μg of pCMV-*hHPSE1* (or pCMV-XL4) and 20 mm Tris-HCl (pH 7.5) was mixed with 47 μl of MultiFectum (Promega). After incubation at room temperature for 30 min, 150 μl of Opti-MEM^TM^ I (Thermo Fisher Scientific) was added. HEK293 cells (8 × 10^5^ cells) in 2 ml of DMEM with 10% FBS were transfected with 200 μl of plasmid/MultiFectum complex in Opti-MEM and cultured in DMEM containing 10% FBS for 24 h. After replacing the culture medium with fresh medium, the cells were cultured with DMEM with 10% FBS. After 24 h, the cells were washed with PBS and lysed with 50 μl of buffer E (0.1 m phosphate buffer, pH 6.0, 0.15 m NaCl, 1 mm phenylmethanesulfonyl fluoride, 10 μg/ml Leupeptin, and 1% Nonidet P-40). HPSE activity in 10 μg of cell lysate proteins was measured using a heparan-degrading enzyme assay kit (TaKaRa Bio) following the supplier's instructions.

### Measurement of scission activity of HPSE and proHPSE by GPC with post-column derivatization

To analyze scission activity of proHPSE in conditioned medium, HEK293 cells were transfected with pCMV-*hHPSE1* as described above. After replacing the culture medium with fresh DMEM (serum-free), the cells were cultured for a further 24 h using DMEM (serum-free) containing 50 μg/ml of unfractionated heparin (New Zealand Pharmaceuticals Ltd., Palmerston North, New Zealand). Thereafter, cultured medium was transferred to clean conical tubes, and the cells were removed completely by centrifugation at 1500 rpm for 10 min. ACR or H_2_O_2_ at the specified concentrations were subsequently added to the resulting conditioned medium (0.5 ml), containing 50 μg/ml of heparin, and further incubated at 37 °C for 24 h. After incubation, 0.5 ml of chloroform/isoamyl alcohol (24:1) was added to the conditioned medium and mixed well to remove proteins. The resulting supernatant was desalted using the Amicon Ultra centrifugal Filter 3K device and lyophilized. Dried samples containing heparin were resuspend with 100 μl of water, and 20 μl (∼5 μg) of heparin solution was submitted to GPC with post-column derivatization. GPC was performed using an Asahipak 510 HQ column (7.6 mm, i.d. × 300 mm) (Showa Denko K. K., Tokyo, Japan). The isocratic elution conditions were as follows: eluent, 10 mm NH_4_HCO_3_; flow rate, 0.3 mL/min. Aqueous (0.5% (w/v)) 2-cyanoacetamide solution and 1 m NaOH were added to the eluent at the same flow rates (0.25 ml/min) using a double plunger pump. Heparin was monitored fluorometrically (excitation, 346 nm; emission, 410 nm).

### Identification of ACR-conjugated amino acid residues in proHPSE

For identification of ACR-conjugated amino acid residues in proHPSE, proHPSE was extracted from the conditioned medium of HEK293 cells transfected with pCMV-*hHPSE1*. Briefly, 30 ml of conditioned medium containing proHPSE and 50 μg/ml heparin was submitted to HiTrap Heparin HP Columns (GE Healthcare Life Science) and equilibrated with 25 mm Tris-HCl (pH 7.5), and eluted proteins were monitored by UV absorbance at 280 nm. ProHPSE was eluted with 1 m NaCl in 25 mm Tris-HCl (pH 7.5) after washing the column with 150 mm and 300 mm NaCl in 25 mm Tris-HCl (pH 7.5). The proHPSE fraction was collected using the Amicon Ultra Centrifugal Filter 3K device, and desalting/buffer exchange was performed using 100 mm NaCl in 25 mm Tris-HCl (pH 7.5). Ten μg of protein in the proHPSE-rich fraction in a buffer containing 100 mm NaCl and 25 mm Tris-HCl (pH 7.5) was exposed to 500 μm ACR and incubated at 37 °C for 24 h. After incubation, proteins were subjected to 10.5% SDS-PAGE and stained with Coomassie Brilliant Blue staining solution. The 65-kDa protein band was excised, reduced with DTT, and alkylated with acrylamide. The protein band was digested with TPCK-treated trypsin (Worthington Biochemical Co, Lakewood, NJ, USA) and PNGase F (Roche Diagnostics, Rotkreuz, Switzerland) at 37 °C overnight. An aliquot of the digestion mixture was analyzed using a Q Exactive mass spectrometer (Thermo Fisher Scientific) coupled with Easy-nLC 1000 (Thermo Fisher Scientific). The peptides were separated using a nano-electrospray ionization column (C18, φ75 μm × 100 mm, 3 μm; Nikkyo Technos, Japan) with a linear gradient of 0–99% buffer (100% acetonitrile and 0.1% formic acid) at a flow rate of 300 nl/min over 20 min. MS (resolution 70,000) and MS/MS (resolution 17,500) data were acquired in a data-dependent TOP10 method. Obtained MS and MS/MS data were quantified using the Proteome Discoverer 2.2 (Thermo Fisher Scientific) with a q-value threshold of 0.05 using MASCOT search engine Version 2.6 (Matrix Science, London, UK) against the in-house database (containing proHPSE), using the following parameters: type of search, MS/MS ion search; enzyme, trypsin; fixed modification, none; variable modifications, Gln→N pyro-Glu (N-term Q), oxidation (M), propionamide (C), deamidated (NQ), ACR adduct (C): C(3) H(4) O(1), ACR adduct (N-term): C(3) H(2), *N*^ε^-(3-formyl-3,4-dehydropiperidino)lysine (K): C(6) H(6) O(1), *N*^ε^-(3-methylpyridinium)lysine (K): C(6) H(4) and Nim-propanal histidine (H): C(3) H(4) O(1); mass values, monoisotopic; peptide mass tolerance, 15 ppm; fragment mass tolerance, ±30 mmu; peptide charge, 1 +, 2 +, and 3 +; maximum missed cleavages, 3; instrument type, ESI-TRAP. ACR conjugated with cysteine is shifted at the N terminus of the peptide by Michael addition, and a Schiff base is formed (51).

### Statistics

Values are indicated as means ± S.D. Normality was evaluated by the D'Agostino and Pearson omnibus normality test. The significance of difference between two groups was analyzed using the Student's *t* test. One-way analysis of variance followed by Dunnett's test was used to evaluate the significance of the difference in groups treated with NAC and/or oligosaccharides of normally distributed variables. The Kruskal-Wallis test was used for nonnormally distributed variables. The statistical calculations were carried out using GraphPad Prism version 8.4.2 (GraphPad Software), RRID:SCR_002798.

## Data availability

MS proteomics data have been deposited to the ProteomeXchange Consortium via the PRIDE partner repository (PXD021040). All data are contained within this article and in the supporting information.
