# Supplementary material for: Ischemic stroke disrupts the endothelial glycocalyx through activation of proHPSE via acrolein exposure
Source: J Biol Chem. 2021 Jan 13;295(52):18614–24. doi: 10.1074/jbc.RA120.015105 (PMC7939480; doi:10.1074/jbc.RA120.015105)
Supplement: Supplementary file 1 [file mmc1.pdf]

Supporting information

Ischemic stroke disrupts the endothelial glycocalyx through activation of proHPSE via acrolein exposure

Kenta Ko[1], Takehiro Suzuki[2], Ryota Ishikawa[1], Natsuko Hattori[1], Risako Ito[3], Kenta Umehara[1], Tomomi Furihata[4], Naoshi Dohmae[2], Robert J. Linhardt[5], Kazuei Igarashi[1, 6], Toshihiko Toida[1] & Kyohei Higashi[3].

[1]*Graduate School of Pharmaceutical Sciences, Chiba University, 1-8-1 Inohana, Chuo-ku, Chiba 260-8675, Japan;* [2]*RIKEN Center for Sustainable Resource Science, 2-1 Hirosawa, Wako, Saitama 351-0198, Japan;* [3]*Faculty of Pharmaceutical Sciences, Tokyo University of Science, 2641 Yamazaki, Noda, Chiba 278-8510, Japan.* [4]*School of Pharmacy, Tokyo University of Pharmacy and Life Sciences, Tokyo 192-0392, Japan;* [5]*Center for Biotechnology and Interdisciplinary Studies, Rensselaer Polytechnic Institute, 110 8th Street Troy, NY 12180, USA;* [6]*Amine Pharma Research Institute, Innovation Plaza at Chiba University, 1-8-15 Inohana, Chuo-ku, Chiba 260-0856, Japan;*

Correspondence: Kyohei Higashi, PhD, Faculty of Pharmaceutical Sciences, Tokyo University of Science, 2641 Yamazaki, Noda, Chiba 278-8510, Japan. E-mail: higase@rs.tus.ac.jp

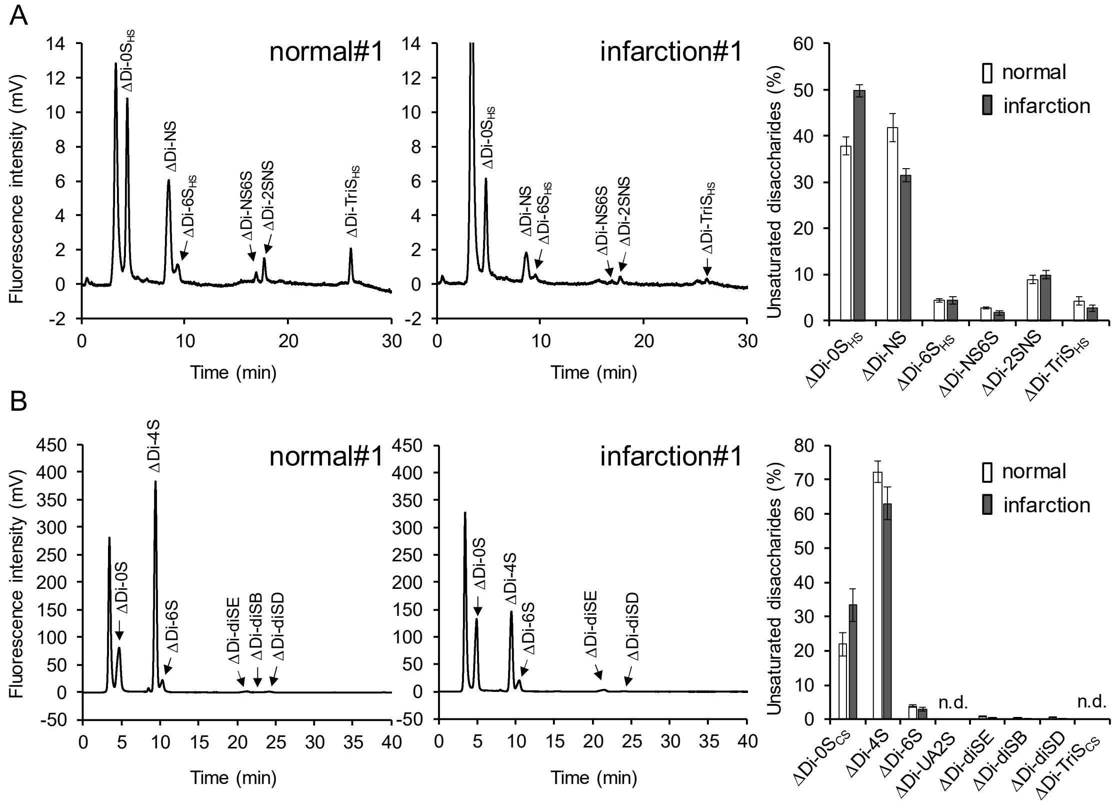

Supplementary Figure 1. Unsaturated disaccharide analysis of HS (A) and CS (B). Extraction of HS and CS from brain tissues, preparation of unsaturated disaccharides, and HPLC were performed as described under Experimental Procedures. Ratios of unsaturated disaccharides are expressed as the mean ± SD (n = 13).

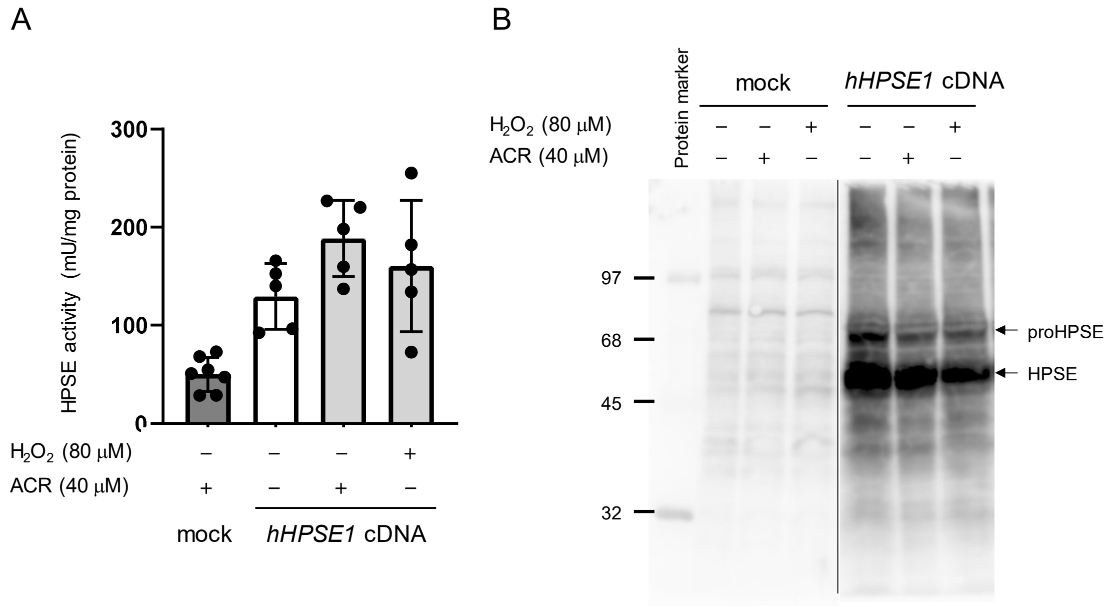

Supplementary Figure 2. Effect of ACR or H_2O_2 on HPSE activity in HEK293 cells transfected with *hHPSE1* cDNA (A) and on the expression levels of HPSE and proHPSE (B). **A,** HPSE activity was measured using a heparan-degrading enzyme assay kit (TaKaRa Bio Inc., Shiga, Japan) following the supplier's instructions. Data are expressed as the mean ± SD. **B**, Western blotting of HPSE and proHPSE was performed using 20 μg of protein extracted from cell lysate. Experiments were repeated three times and reproducible results were obtained.

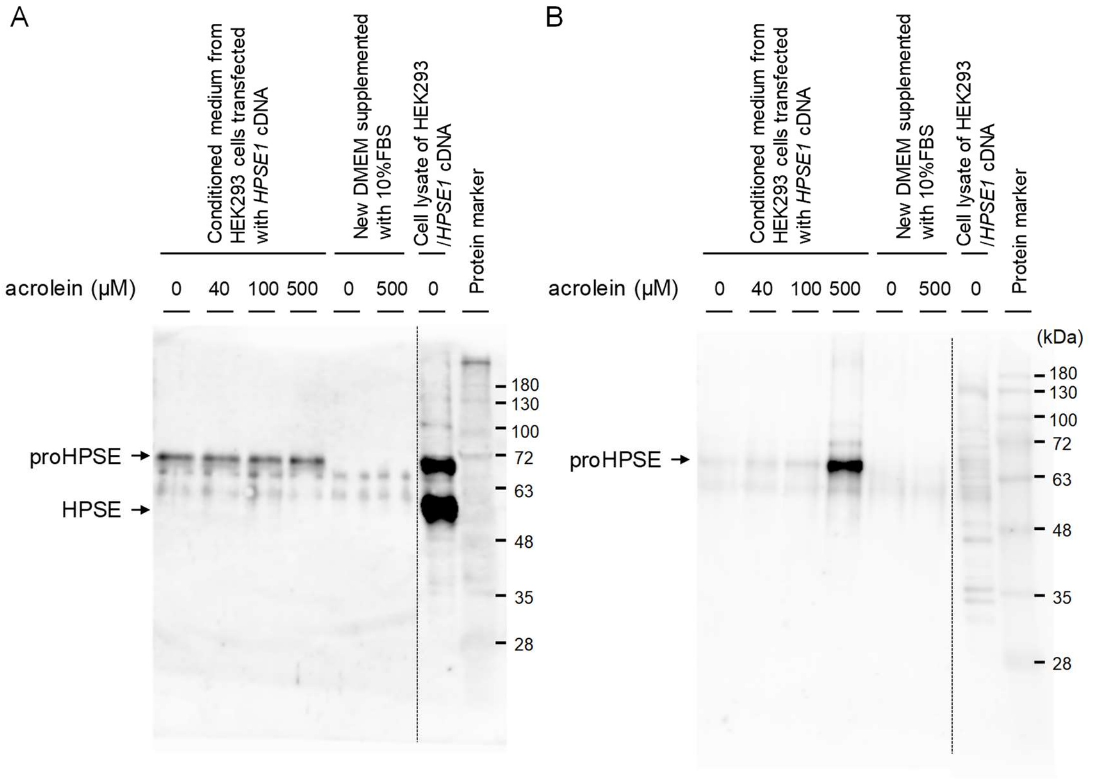

Supplementary Figure 3. Western blotting of proHPSE (A) and ACR-modified proHPSE in conditioned medium. **A**, Western blotting of proHPSE and HPSE was performed using primary antibody of HPSE recognizing both 50-kDa and 8-kDa subunits, respectively. **B**, Western blotting of ACR-modified protein in conditioned medium. Note that proHPSE, but not HPSE, was observed as an ACR-modified protein in conditioned medium. For western blotting, 50 μL of conditioned medium was used. Experiments were repeated three times and reproducible results were obtained.

Supplementary Figure 4-A

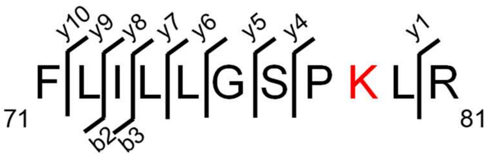

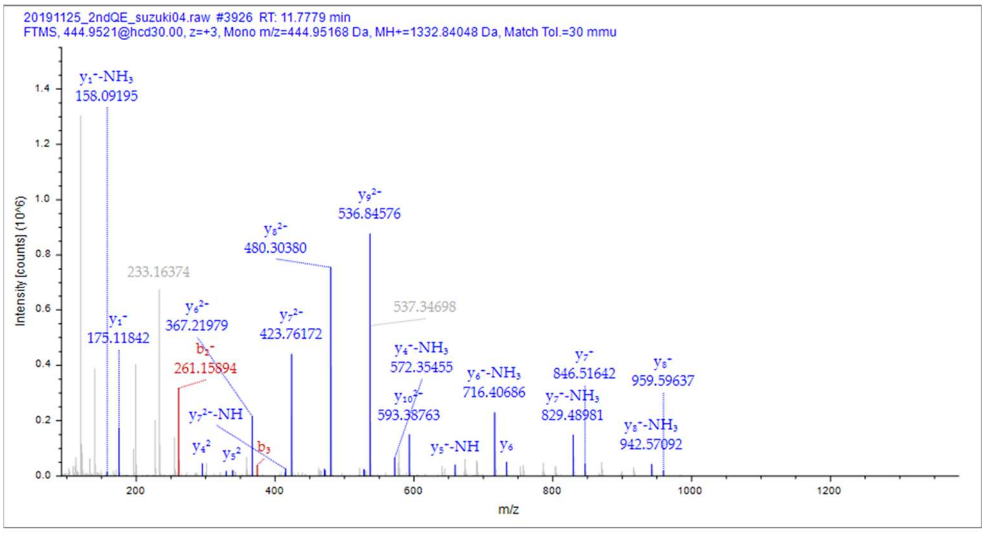

#1	b⁺	b²⁺	b³⁺	Seq.	y⁺	y²⁺	y³⁺	#2
1	148.07569	74.54148	50.03008	F				11
2	261.15975	131.08352	87.72477	L	1185.77179	593.38953	395.92878	10
3	374.24382	187.62555	125.41946	I	1072.68773	536.84750	358.23409	9
4	487.32788	244.16758	163.11415	L	959.60366	480.30547	320.53941	8
5	600.41195	300.70961	200.80883	L	846.51960	423.76344	282.84472	7
6	657.43341	329.22034	219.81599	G	733.43553	367.22141	245.15003	6
7	744.46544	372.73636	248.82666	S	676.41407	338.71067	226.14287	5
8	841.51820	421.26274	281.17759	P	589.38204	295.19466	197.13220	4
9	1045.64447	523.32587	349.21967	K-Delta:H(4)C(6)	492.32928	246.66828	164.78128	3
10	1158.72853	579.86790	386.91436	L	288.20302	144.60515	96.73919	2
11				R	175.11895	88.06311	59.04450	1

Supplementary Figure 4-B

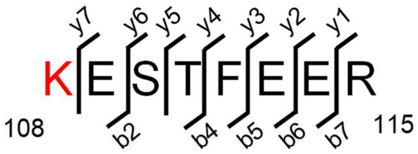

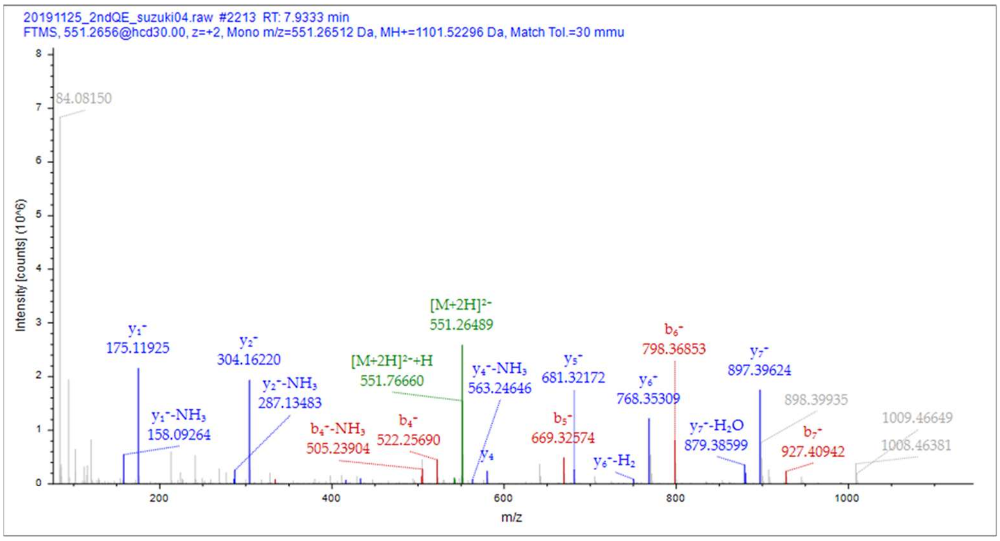

#1	b^+	b^{2+}	Seq.	y^+	y^{2+}	#2
1	205.13354	103.07041	K-Delta:H(4)C(6)			8
2	334.17613	167.59170	E	897.39485	449.20106	7
3	421.20816	211.10772	S	768.35226	384.67977	6
4	522.25584	261.63156	T	681.32023	341.16375	5
5	669.32425	335.16576	F	580.27255	290.63991	4
6	798.36685	399.68706	E	433.20414	217.10571	3
7	927.40944	464.20836	E	304.16155	152.58441	2
8			R	175.11895	88.06311	1

Supplementary Figure 4-C

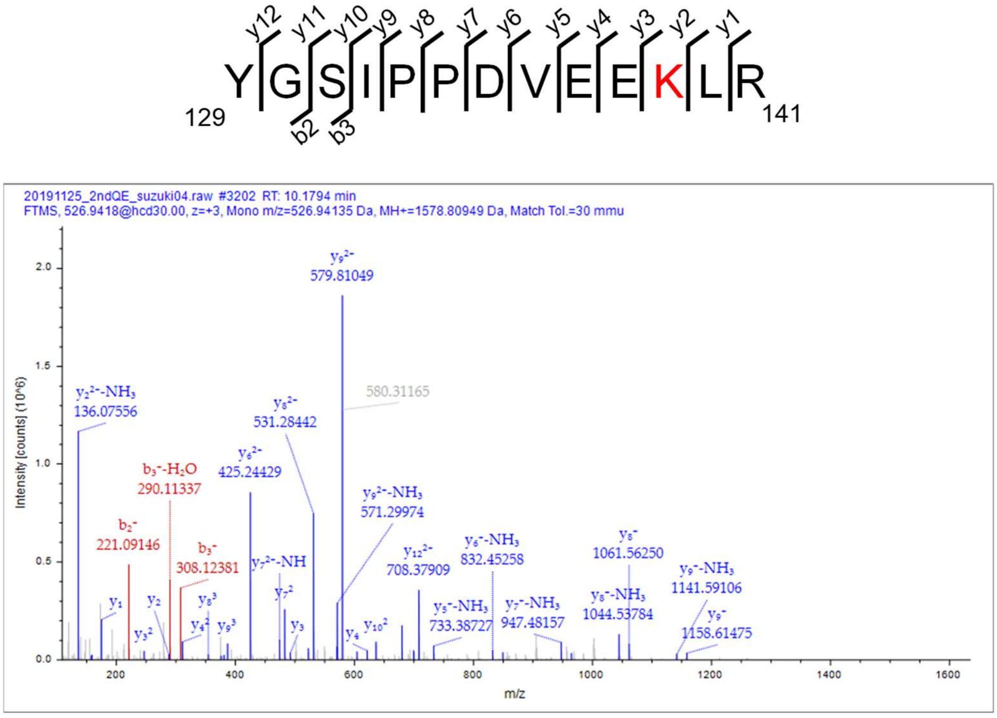

#1	b⁺	b²⁺	b³⁺	Seq.	y⁺	y²⁺	y³⁺	#2
1	164.07061	82.53894	55.36172	Y				13
2	221.09207	111.04967	74.36887	G	1415.75291	708.38009	472.58915	12
3	308.12410	154.56569	103.37955	S	1358.73144	679.86936	453.58200	11
4	421.20816	211.10772	141.07424	I	1271.69941	636.35335	424.57132	10
5	518.26092	259.63410	173.42516	P	1158.61535	579.81131	386.87663	9
6	615.31369	308.16048	205.77608	P	1061.56259	531.28493	354.52571	8
7	730.34063	365.67395	244.11839	D	964.50982	482.75855	322.17479	7
8	829.40905	415.20816	277.14120	V	849.48288	425.24508	283.83248	6
9	958.45164	479.72946	320.15540	E	750.41447	375.71087	250.80967	5
10	1087.49423	544.25075	363.16959	E	621.37187	311.18957	207.79548	4
11	1291.62049	646.31389	431.21168	K-Delta:H(4)C(6)	492.32928	246.66828	164.78128	3
12	1404.70456	702.85592	468.90637	L	288.20302	144.60515	96.73919	2
13				R	175.11895	88.06311	59.04450	1

Supplementary Figure 4-D

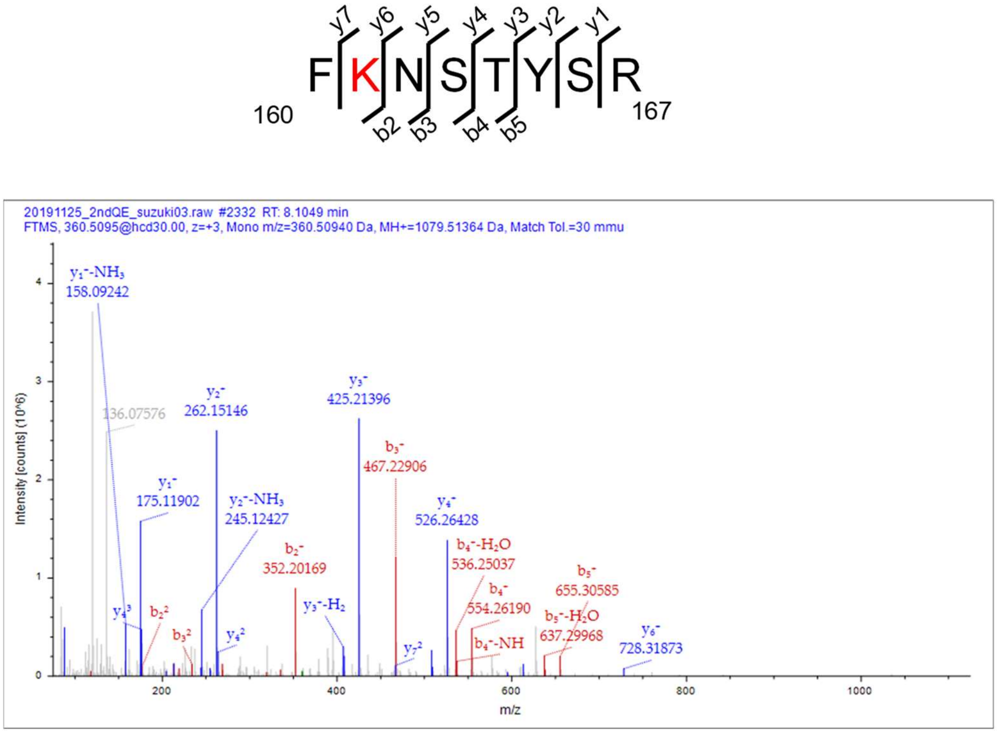

#1	b⁺	b²⁺	b³⁺	Seq.	y⁺	y²⁺	y³⁺	#2
1	148.07569	74.54148	50.03008	F				8
2	352.20195	176.60462	118.07217	K-Delta:H(4)C(6)	932.44722	466.72725	311.48726	7
3	467.22890	234.11809	156.41448	N-Deamidated	728.32096	364.66412	243.44517	6
4	554.26093	277.63410	185.42516	S	613.29402	307.15065	205.10286	5
5	655.30860	328.15794	219.10772	T	526.26199	263.63463	176.09218	4
6	818.37193	409.68960	273.46216	Y	425.21431	213.11079	142.40962	3
7	905.40396	453.20562	302.47284	S	262.15098	131.57913	88.05518	2
8				R	175.11895	88.06311	59.04450	1

Supplementary Figure 4-E

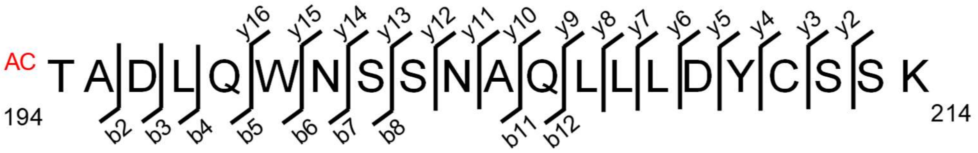

AC: Acrolein adduct (N-terminal)

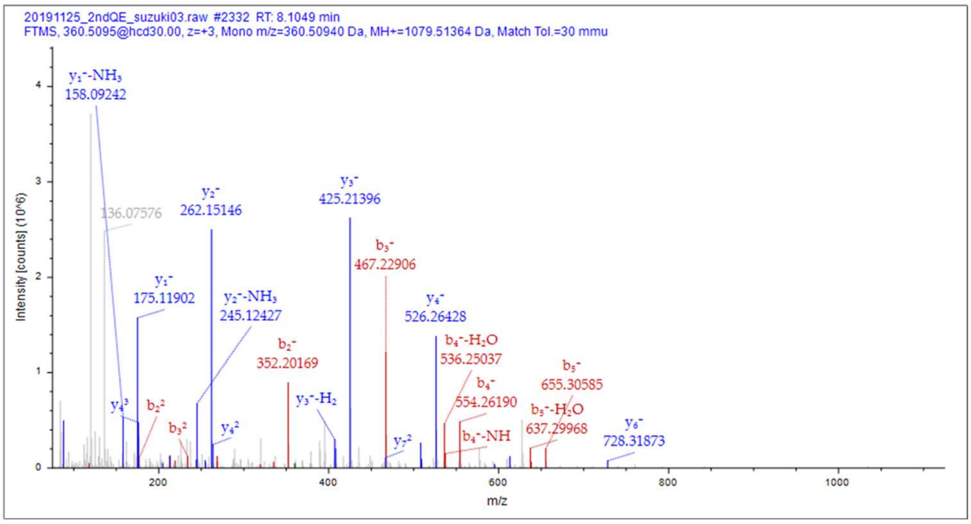

#1	b⁺	b²⁺	Seq.	y⁺	y²⁺	#2
1	140.07061	70.53894	T-Acrolein adduct2			21
2	211.10772	106.05750	A	2257.04414	1129.02571	20
3	326.13466	163.57097	D	2186.00702	1093.50715	19
4	439.21873	220.11300	L	2070.98008	1035.99368	18
5	567.27730	284.14229	Q	1957.89602	979.45165	17
6	753.35662	377.18195	W	1829.83744	915.42236	16
7	868.38356	434.69542	N-Deamidated	1643.75813	822.38270	15
8	955.41559	478.21143	S	1528.73118	764.86923	14
9	1042.44762	521.72745	S	1441.69916	721.35322	13
10	1156.49054	578.74891	N	1354.66713	677.83720	12
11	1227.52766	614.26747	A	1240.62420	620.81574	11
12	1355.58623	678.29676	Q	1169.58709	585.29718	10
13	1468.67030	734.83879	L	1041.52851	521.26789	9
14	1581.75436	791.38082	L	928.44444	464.72586	8
15	1694.83843	847.92285	L	815.36038	408.18383	7
16	1809.86537	905.43632	D	702.27632	351.64180	6
17	1972.92870	986.96799	Y	587.24937	294.12833	5
18	2075.93788	1038.47258	C	424.18605	212.59666	4
19	2162.96991	1081.98859	S	321.17686	161.09207	3
20	2250.00194	1125.50461	S	234.14483	117.57605	2
21			K	147.11280	74.06004	1

Supplementary Figure 4-F

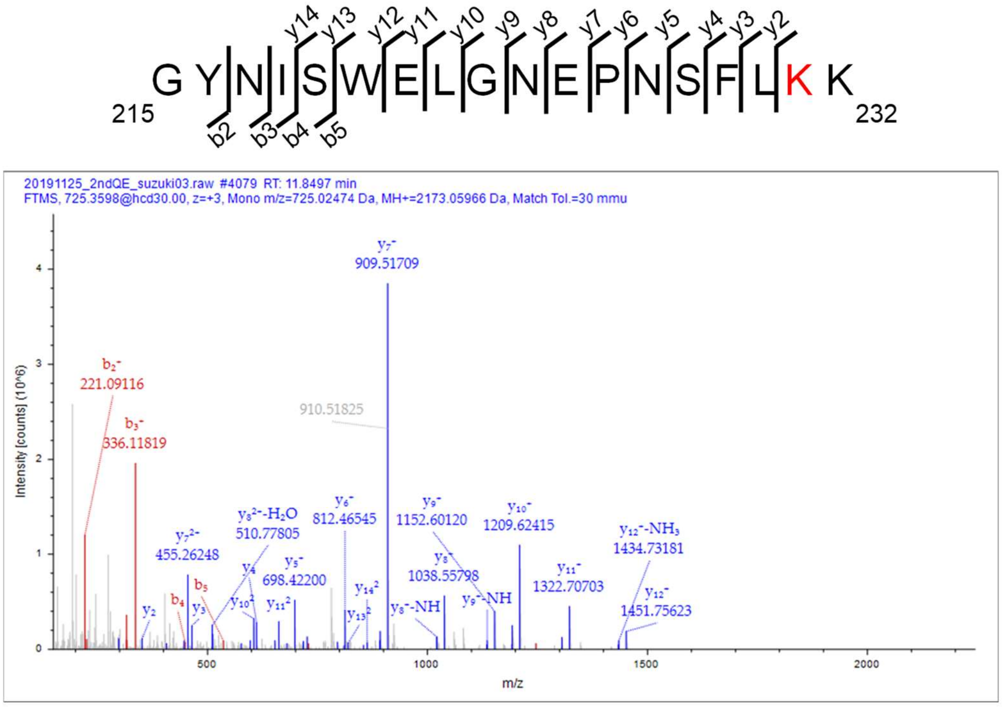

#1	b⁺	b²⁺	b³⁺	Seq.	y⁺	y²⁺	y³⁺	#2
1	58.02874	29.51801	20.01443	G				18
2	221.09207	111.04967	74.36887	Y	2116.03858	1058.52293	706.01771	17
3	336.11901	168.56314	112.71119	N-Deamidated	1952.97525	976.99127	651.66327	16
4	449.20308	225.10518	150.40588	I	1837.94831	919.47779	613.32095	15
5	536.23510	268.62119	179.41655	S	1724.86425	862.93576	575.62627	14
6	722.31442	361.66085	241.44299	W	1637.83222	819.41975	546.61559	13
7	851.35701	426.18214	284.45719	E	1451.75291	726.38009	484.58915	12
8	964.44107	482.72418	322.15188	L	1322.71031	661.85879	441.57496	11
9	1021.46254	511.23491	341.15903	G	1209.62625	605.31676	403.88027	10
10	1135.50547	568.25637	379.17334	N	1152.60478	576.80603	384.87311	9
11	1264.54806	632.77767	422.18754	E	1038.56186	519.78457	346.85880	8
12	1361.60082	681.30405	454.53846	P	909.51926	455.26327	303.84461	7
13	1475.64375	738.32551	492.55277	N	812.46650	406.73689	271.49368	6
14	1562.67578	781.84153	521.56344	S	698.42357	349.71542	233.47938	5
15	1709.74419	855.37573	570.58625	F	611.39155	306.19941	204.46870	4
16	1822.82826	911.91777	608.28094	L	464.32313	232.66520	155.44589	3
17	2026.95452	1013.98090	676.32302	K-Delta:H(4)C(6)	351.23907	176.12317	117.75121	2
18				K	147.11280	74.06004	49.70912	1

Supplementary Figure 4-G

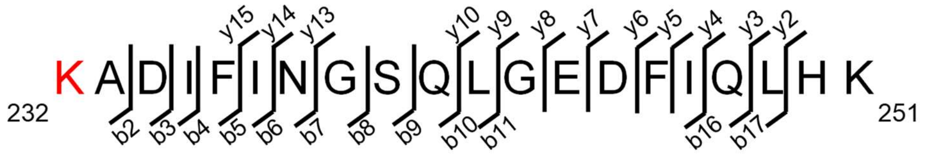

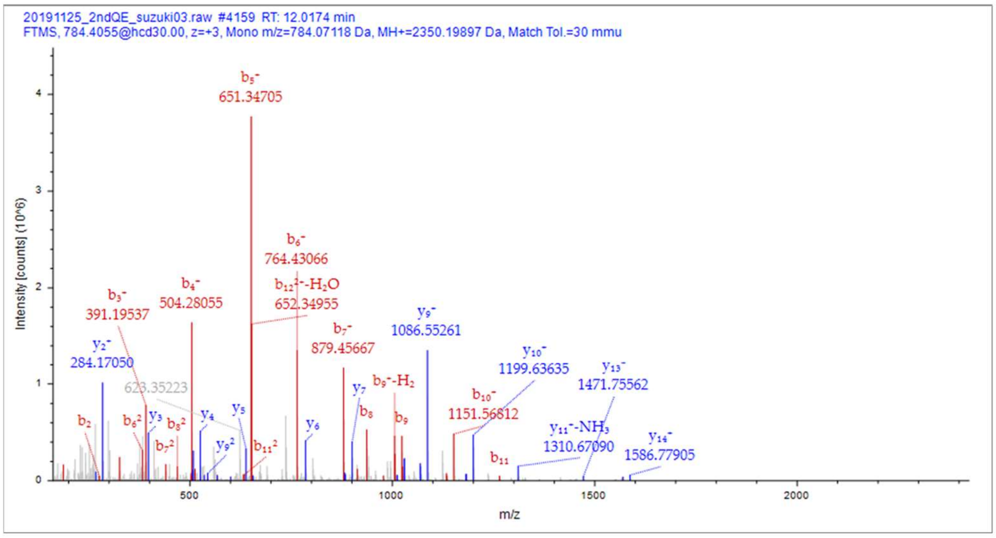

#1	b⁺	b²⁺	b³⁺	Seq.	y⁺	y²⁺	y³⁺	#2
1	205.13354	103.07041	69.04936	K-Delta:H(4)C(6)				20
2	276.17065	138.58896	92.72840	A	2146.08151	1073.54439	716.03202	19
3	391.19760	196.10244	131.07072	D	2075.04440	1038.02584	692.35298	18
4	504.28166	252.64447	168.76540	I	1960.01745	980.51237	654.01067	17
5	651.35007	326.17868	217.78821	F	1846.93339	923.97033	616.31598	16
6	764.43414	382.72071	255.48290	I	1699.86498	850.43613	567.29318	15
7	879.46108	440.23418	293.82521	N-Deamidated	1586.78091	793.89409	529.59849	14
8	936.48255	468.74491	312.83237	G	1471.75397	736.38062	491.25617	13
9	1023.51457	512.26093	341.84304	S	1414.73250	707.86989	472.24902	12
10	1151.57315	576.29021	384.52923	Q	1327.70048	664.35388	443.23834	11
11	1264.65721	632.83225	422.22392	L	1199.64190	600.32459	400.55215	10
12	1321.67868	661.34298	441.23108	G	1086.55783	543.78256	362.85746	9
13	1450.72127	725.86427	484.24527	E	1029.53637	515.27182	343.85031	8
14	1565.74821	783.37775	522.58759	D	900.49378	450.75053	300.83611	7
15	1712.81663	856.91195	571.61039	F	785.46684	393.23706	262.49380	6
16	1825.90069	913.45398	609.30508	I	638.39842	319.70285	213.47099	5
17	1953.95927	977.48327	651.99127	Q	525.31436	263.16082	175.77630	4
18	2067.04333	1034.02531	689.68596	L	397.25578	199.13153	133.09011	3
19	2204.10225	1102.55476	735.37227	H	284.17172	142.58950	95.39542	2
20				K	147.11280	74.06004	49.70912	1

Supplementary Figure 4-H

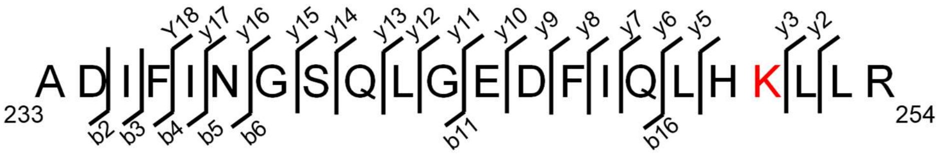

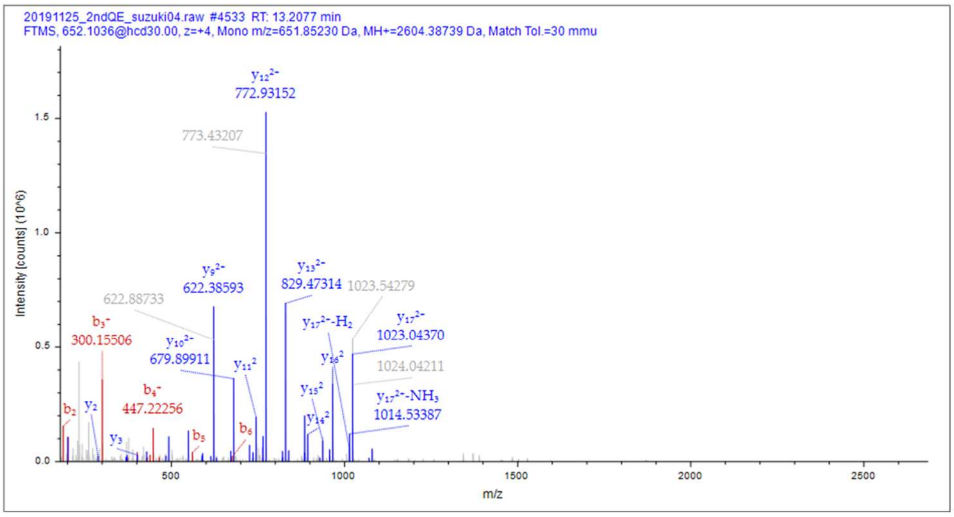

#1	b⁺	b²⁺	b³⁺	b⁴⁺	Seq.	y⁺	y²⁺	y³⁺	y⁴⁺	#2
1	72.04439	36.52583	24.68631	18.76656	A					22
2	187.07133	94.03930	63.02863	47.52329	D	2533.34494	1267.17611	845.11983	634.09169	21
3	300.15540	150.58134	100.72332	75.79431	I	2418.31799	1209.66263	806.77752	605.33496	20
4	447.22381	224.11554	149.74612	112.56141	F	2305.23393	1153.12060	769.08283	577.06394	19
5	560.30788	280.65758	187.44081	140.83243	I	2158.16551	1079.58640	720.06002	540.29684	18
6	675.33482	338.17105	225.78312	169.58916	N-Deamidated	2045.08145	1023.04436	682.36533	512.02582	17
7	732.35628	366.68178	244.79028	183.84453	G	1930.05451	965.53089	644.02302	483.26908	16
8	819.38831	410.19779	273.80095	205.60254	S	1873.03304	937.02016	625.01587	469.01372	15
9	947.44689	474.22708	316.48715	237.61718	Q	1786.00102	893.50415	596.00519	447.25571	14
10	1060.53095	530.76911	354.18184	265.88820	L	1657.94244	829.47486	553.31900	415.24107	13
11	1117.55242	559.27985	373.18899	280.14356	G	1544.85837	772.93283	515.62431	386.97005	12
12	1246.59501	623.80114	416.20319	312.40421	E	1487.83691	744.42209	496.61715	372.71468	11
13	1361.62195	681.31461	454.54550	341.16095	D	1358.79432	679.90080	453.60296	340.45404	10
14	1508.69037	754.84882	503.56831	377.92805	F	1243.76737	622.38733	415.26064	311.69730	9
15	1621.77443	811.39085	541.26299	406.19906	I	1096.69896	548.85312	366.23784	274.93020	8
16	1749.83301	875.42014	583.94919	438.21371	Q	983.61490	492.31109	328.54315	246.65918	7
17	1862.91707	931.96217	621.64387	466.48473	L	855.55632	428.28180	285.85696	214.64454	6
18	1999.97598	1000.49163	667.33018	500.74945	H	742.47225	371.73977	248.16227	186.37352	5
19	2204.10225	1102.55476	735.37227	551.78102	K-Delta:H(4)C(6)	605.41334	303.21031	202.47597	152.10879	4
20	2317.18631	1159.09679	773.06695	580.05203	L	401.28708	201.14718	134.43388	101.07723	3
21	2430.27037	1215.63882	810.76164	608.32305	L	288.20302	144.60515	96.73919	72.80621	2
22					R	175.11895	88.06311	59.04450	44.53520	1

Supplementary Figure 4-I

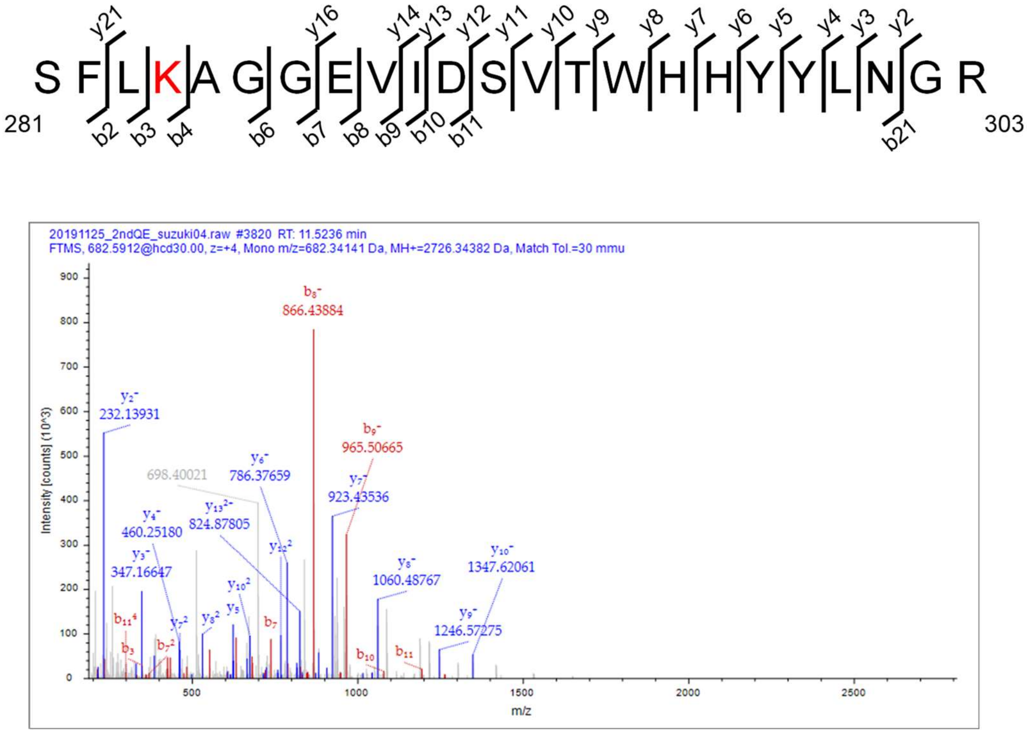

#1	b^{+}	b^{2+}	b^{3+}	b^{4+}	Seq.	y^{+}	y^{2+}	y^{3+}	y^{4+}	#2
1	88.03930	44.52329	30.01795	22.76528	S					23
2	235.10772	118.05750	79.04076	59.53239	F	2639.30413	1320.15570	880.43956	660.58149	22
3	348.19178	174.59953	116.73545	87.80340	L	2492.23572	1246.62150	831.41676	623.81439	21
4	552.31805	276.66266	184.77753	138.83497	K-Delta:H(4)C(6)	2379.15166	1190.07947	793.72207	595.54337	20
5	623.35516	312.18122	208.45657	156.59425	A	2175.02539	1088.01633	725.67998	544.51181	19
6	680.37662	340.69195	227.46373	170.84961	G	2103.98828	1052.49778	702.00094	526.75253	18
7	737.39809	369.20268	246.47088	185.10498	G	2046.96681	1023.98705	682.99379	512.49716	17
8	866.44068	433.72398	289.48508	217.36563	E	1989.94535	995.47631	663.98663	498.24180	16
9	965.50909	483.25819	322.50788	242.13273	V	1860.90276	930.95502	620.97244	465.98115	15
10	1078.59316	539.80022	360.20257	270.40375	I	1761.83434	881.42081	587.94963	441.21404	14
11	1193.62010	597.31369	398.54488	299.16048	D	1648.75028	824.87878	550.25494	412.94303	13
12	1280.65213	640.82970	427.55556	320.91849	S	1533.72334	767.36531	511.91263	384.18629	12
13	1379.72054	690.36391	460.57837	345.68559	V	1446.69131	723.84929	482.90195	362.42828	11
14	1480.76822	740.88775	494.26092	370.94751	T	1347.62290	674.31509	449.87915	337.66118	10
15	1666.84753	833.92741	556.28736	417.46734	W	1246.57522	623.79125	416.19659	312.39926	9
16	1803.90645	902.45686	601.97367	451.73207	H	1060.49590	530.75159	354.17015	265.87943	8
17	1940.96536	970.98632	647.65997	485.99680	H	923.43699	462.22213	308.48385	231.61471	7
18	2104.02869	1052.51798	702.01441	526.76263	Y	786.37808	393.69268	262.79754	197.34998	6
19	2267.09201	1134.04965	756.36886	567.52846	Y	623.31475	312.16101	208.44310	156.58415	5
20	2380.17608	1190.59168	794.06354	595.79948	L	460.25142	230.62935	154.08866	115.81831	4
21	2495.20302	1248.10515	832.40586	624.55621	N-Deamidated	347.16736	174.08732	116.39397	87.54730	3
22	2552.22449	1276.61588	851.41301	638.81158	G	232.14042	116.57385	78.05166	58.79056	2
23					R	175.11895	88.06311	59.04450	44.53520	1

Supplementary Figure 4-J

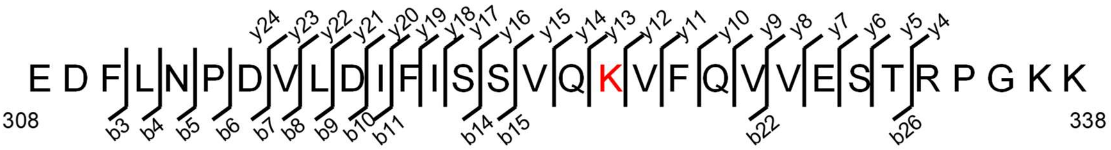

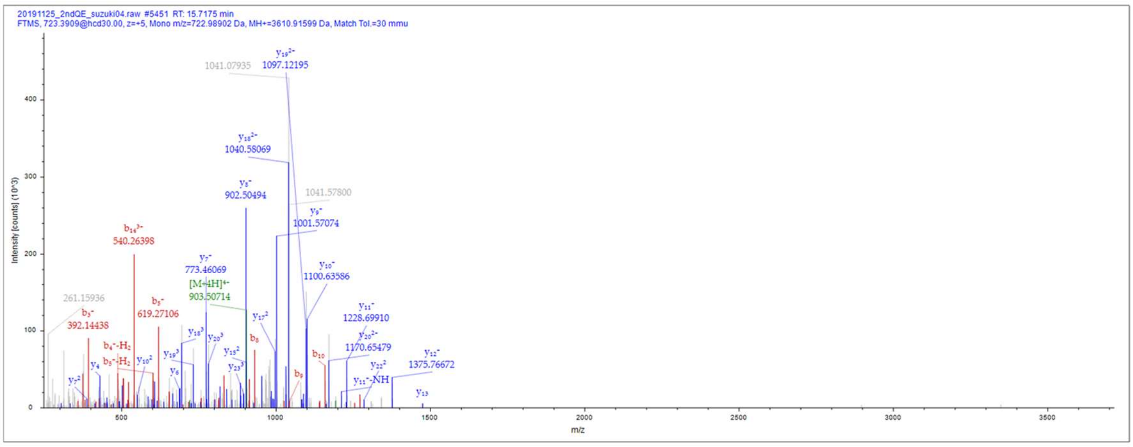

#1	b+	b2+	b3+	b4+	b5+	Seq.	y+	y2+	y3+	y4+	y5+	#2
1	130.04987	65.52857	44.02147	33.26792	26.81580	E						31
2	245.07681	123.04204	82.36379	62.02466	49.82118	D	3481.87297	1741.44012	1161.29584	871.22370	697.18041	30
3	392.14523	196.57625	131.38659	98.79176	79.23487	F	3366.84602	1683.92665	1122.95353	842.46696	674.17503	29
4	505.22929	253.11828	169.08128	127.06278	101.85168	L	3219.77761	1610.39244	1073.93072	805.69986	644.76134	28
5	619.27222	310.13975	207.09559	155.57351	124.66026	N	3106.69355	1553.85041	1036.23603	777.42884	622.14453	27
6	716.32498	358.66613	239.44651	179.83670	144.07082	P	2992.65062	1496.82895	998.22172	748.91811	599.33595	26
7	831.35192	416.17960	277.78883	208.59344	167.07621	D	2895.59785	1448.30257	965.87080	724.65492	579.92539	25
8	930.42034	465.71381	310.81163	233.36054	186.88989	V	2780.57091	1390.78909	927.52849	695.89819	556.92000	24
9	1043.50440	522.25584	348.50632	261.63156	209.50670	L	2681.50250	1341.25489	894.50568	671.13108	537.10632	23
10	1158.53135	579.76931	386.84863	290.38829	232.51209	D	2568.41843	1284.71286	856.81100	642.86007	514.48951	22
11	1271.61541	636.31134	424.54332	318.65931	255.12890	I	2453.39149	1227.19938	818.46868	614.10333	491.48412	21
12	1418.68382	709.84555	473.56613	355.42641	284.54259	F	2340.30743	1170.65735	780.77399	585.83231	468.86731	20
13	1531.76789	766.38758	511.26081	383.69743	307.15940	I	2193.23901	1097.12314	731.75119	549.06521	439.45362	19
14	1618.79992	809.90360	540.27149	405.45544	324.56580	S	2080.15495	1040.58111	694.05650	520.79419	416.83681	18
15	1705.83194	853.41961	569.28217	427.21344	341.97221	S	1993.12292	997.06510	665.04582	499.03619	399.43041	17
16	1804.90036	902.95382	602.30497	451.98055	361.78589	V	1906.09089	953.54908	636.03515	477.27818	382.02400	16
17	1932.95894	966.98311	644.99116	483.99519	387.39761	Q	1807.02248	904.01488	603.01234	452.51108	362.21032	15
18	2137.08520	1069.04624	713.03325	535.02676	428.22286	K-Delta:H(4)C(6)	1678.96390	839.98559	560.32615	420.49643	336.59860	14
19	2236.15361	1118.58044	746.05606	559.79386	448.03654	V	1474.83764	737.92246	492.28406	369.46487	295.77335	13
20	2383.22203	1192.11465	795.07886	596.56096	477.45023	F	1375.76922	688.38825	459.26126	344.69776	275.95967	12
21	2511.28060	1256.14394	837.76505	628.57561	503.06194	Q	1228.70081	614.85404	410.23845	307.93066	246.54598	11
22	2610.34902	1305.67815	870.78786	653.34271	522.87562	V	1100.64223	550.82475	367.55226	275.91602	220.93427	10
23	2709.41743	1355.21235	903.81066	678.10982	542.68931	V	1001.57382	501.29055	334.52946	251.14891	201.12059	9
24	2838.46002	1419.73365	946.82486	710.37046	568.49783	E	902.50541	451.75634	301.50665	226.38181	181.30690	8
25	2925.49205	1463.24966	975.83554	732.12847	585.90423	S	773.46281	387.23504	258.49246	194.12116	155.49838	7
26	3026.53973	1513.77350	1009.51809	757.39039	606.11377	T	686.43078	343.71903	229.48178	172.36315	138.09198	6
27	3182.64084	1591.82406	1061.55180	796.41567	637.33399	R	585.38311	293.19519	195.79922	147.10123	117.88244	5
28	3279.69361	1640.35044	1093.90272	820.67886	656.74454	P	429.28199	215.14464	143.76552	108.07596	86.66222	4
29	3336.71507	1668.86117	1112.90987	834.93422	668.14884	G	332.22923	166.61825	111.41459	83.81277	67.25167	3
30	3464.81003	1732.90865	1155.60820	866.95797	693.76783	K	275.20777	138.10752	92.40744	69.55740	55.84737	2
31						K	147.11280	74.06004	49.70912	37.53366	30.22838	1

Supplementary Figure 4-K

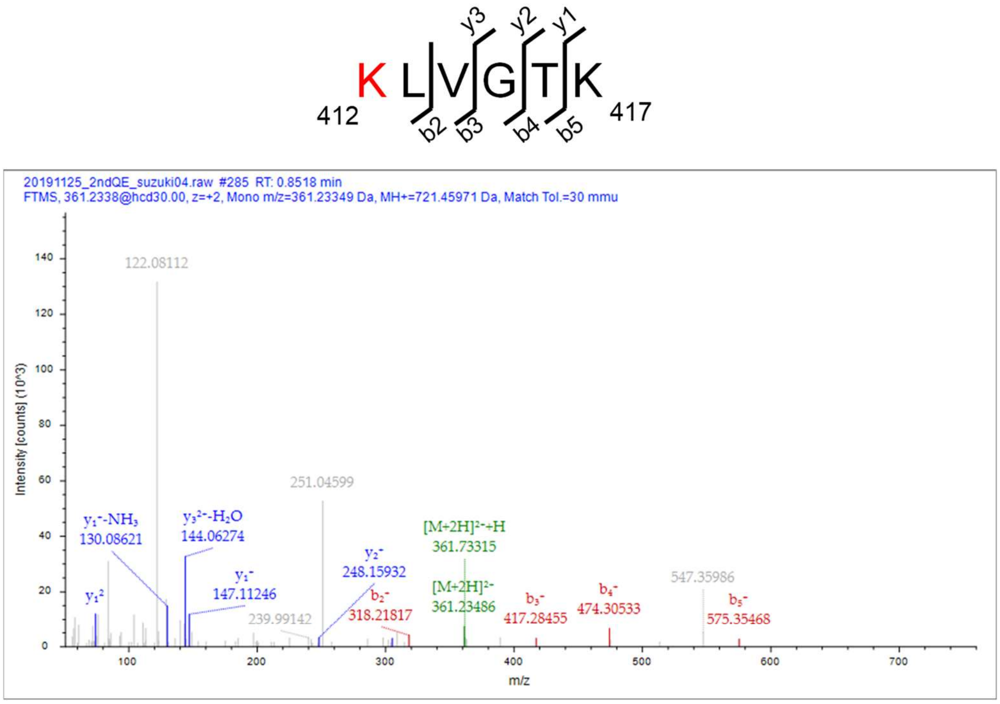

#1	b^+	b^{2+}	Seq.	y^+	y^{2+}	#2
1	205.13354	103.07041	K-Delta:H(4)C(6)			6
2	318.21760	159.61244	L	517.33442	259.17085	5
3	417.28602	209.14665	V	404.25036	202.62882	4
4	474.30748	237.65738	G	305.18195	153.09461	3
5	575.35516	288.18122	T	248.16048	124.58388	2
6			K	147.11280	74.06004	1

Supplementary Figure 4-L

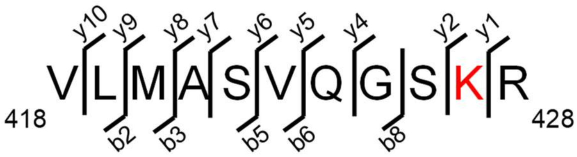

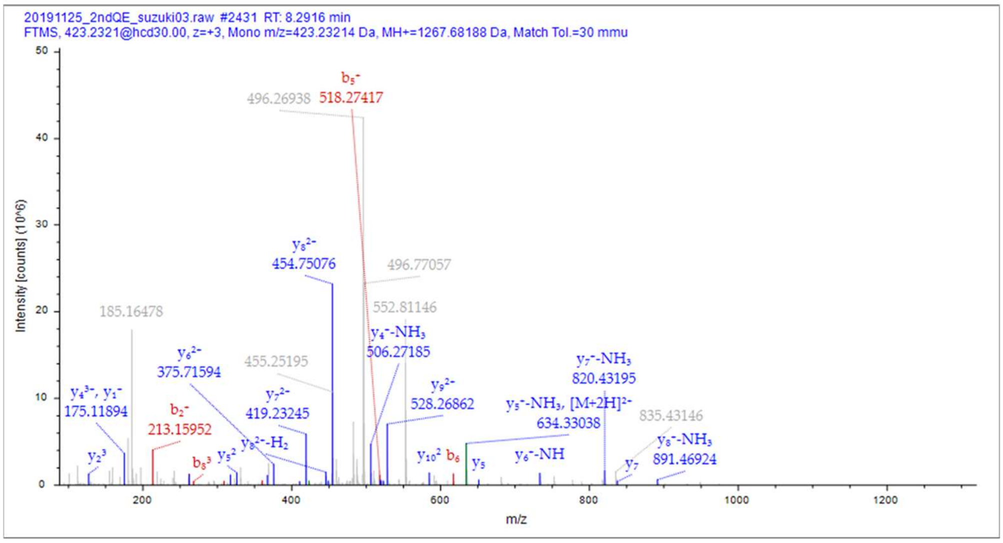

#1	b⁺	b²⁺	b³⁺	Seq.	y⁺	y²⁺	y³⁺	#2
1	100.07569	50.54148	34.03008	V				11
2	213.15975	107.08352	71.72477	L	1168.61430	584.81079	390.20962	10
3	360.19515	180.60122	120.73657	M-Oxidation	1055.53024	528.26876	352.51493	9
4	431.23227	216.11977	144.41561	A	908.49484	454.75106	303.50313	8
5	518.26430	259.63579	173.42628	S	837.45773	419.23250	279.82409	7
6	617.33271	309.16999	206.44909	V	750.42570	375.71649	250.81342	6
7	745.39129	373.19928	249.13528	Q	651.35728	326.18228	217.79061	5
8	802.41275	401.71001	268.14243	G	523.29871	262.15299	175.10442	4
9	889.44478	445.22603	297.15311	S	466.27724	233.64226	156.09727	3
10	1093.57104	547.28916	365.19520	K-Delta:H(4)C(6)	379.24522	190.12625	127.08659	2
11				R	175.11895	88.06311	59.04450	1

Supplementary Figure 4-M

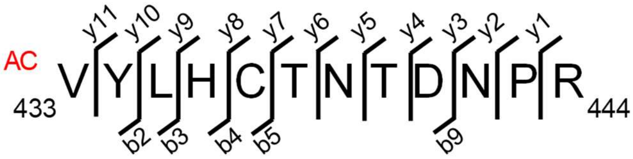

AC: Acrolein adduct (N-terminal)

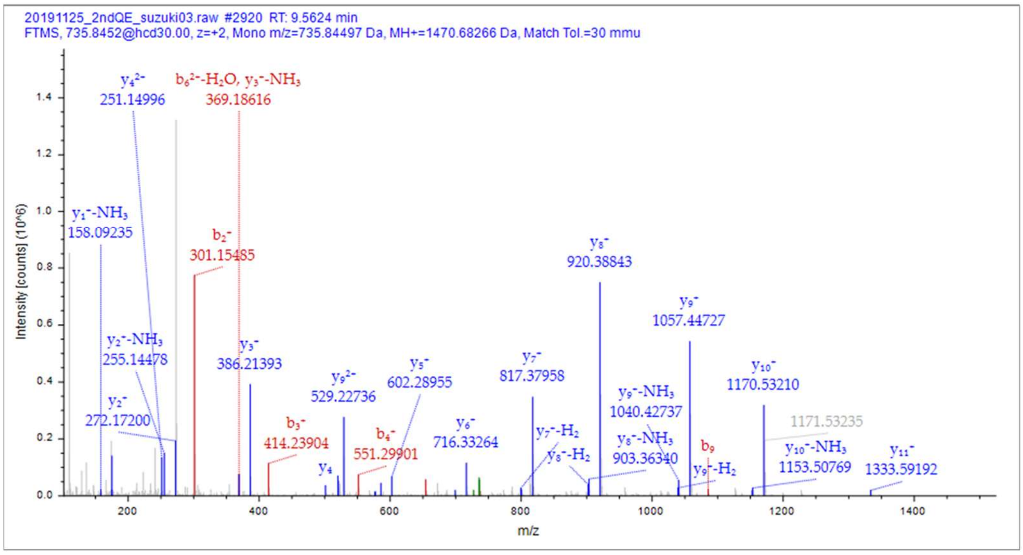

#1	b⁺	b²⁺	Seq.	y⁺	y²⁺	#2
1	138.09134	69.54931	V-Acrolein adduct2			12
2	301.15467	151.08097	Y	1333.59536	667.30132	11
3	414.23873	207.62300	L	1170.53203	585.76965	10
4	551.29764	276.15246	H	1057.44797	529.22762	9
5	654.30683	327.65705	C	920.38906	460.69817	8
6	755.35451	378.18089	T	817.37987	409.19357	7
7	869.39743	435.20236	N	716.33219	358.66973	6
8	970.44511	485.72619	T	602.28926	301.64827	5
9	1085.47206	543.23967	D	501.24159	251.12443	4
10	1199.51498	600.26113	N	386.21464	193.61096	3
11	1296.56775	648.78751	P	272.17172	136.58950	2
12			R	175.11895	88.06311	1

Supplementary Figure 4-N

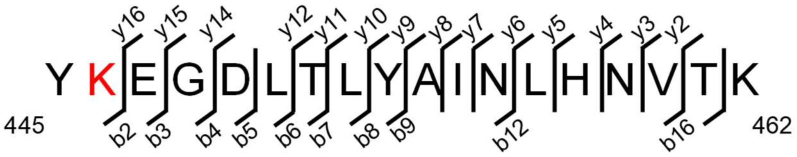

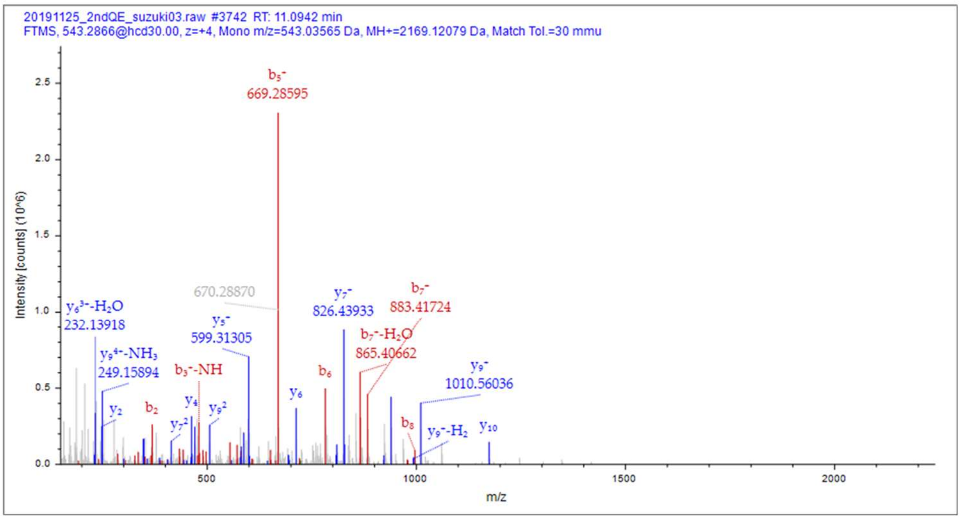

#1	b⁺	b²⁺	b³⁺	b⁴⁺	Seq.	y⁺	y²⁺	y³⁺	y⁴⁺	#2
1	164.07061	82.53894	55.36172	41.77311	Y					18
2	368.19687	184.60207	123.40381	92.80467	K-Delta:H(4)C(6)	2006.05932	1003.53330	669.35796	502.27029	17
3	497.23946	249.12337	166.41800	125.06532	E	1801.93306	901.47017	601.31587	451.23872	16
4	554.26092	277.63410	185.42516	139.32069	G	1672.89046	836.94887	558.30167	418.97807	15
5	669.28787	335.14757	223.76747	168.07742	D	1615.86900	808.43814	539.29452	404.72271	14
6	782.37193	391.68960	261.46216	196.34844	L	1500.84206	750.92467	500.95220	375.96597	13
7	883.41961	442.21344	295.14472	221.61036	T	1387.75799	694.38263	463.25751	347.69496	12
8	996.50367	498.75548	332.83941	249.88138	L	1286.71031	643.85879	429.57496	322.43304	11
9	1159.56700	580.28714	387.19385	290.64721	Y	1173.62625	587.31676	391.88027	294.16202	10
10	1230.60412	615.80570	410.87289	308.40649	A	1010.56292	505.78510	337.52582	253.39619	9
11	1343.68818	672.34773	448.56758	336.67750	I	939.52581	470.26654	313.84679	235.63691	8
12	1457.73111	729.36919	486.58189	365.18823	N	826.44174	413.72451	276.15210	207.36589	7
13	1570.81517	785.91122	524.27657	393.45925	L	712.39882	356.70305	238.13779	178.85516	6
14	1707.87408	854.44068	569.96288	427.72398	H	599.31475	300.16101	200.44310	150.58415	5
15	1822.90103	911.95415	608.30519	456.48071	N-Deamidated	462.25584	231.63156	154.75680	116.31942	4
16	1921.96944	961.48836	641.32800	481.24782	V	347.22890	174.11809	116.41448	87.56268	3
17	2023.01712	1012.01220	675.01056	506.50974	T	248.16048	124.58388	83.39168	62.79558	2
18					K	147.11280	74.06004	49.70912	37.53366	1

Supplementary Figure 4-O

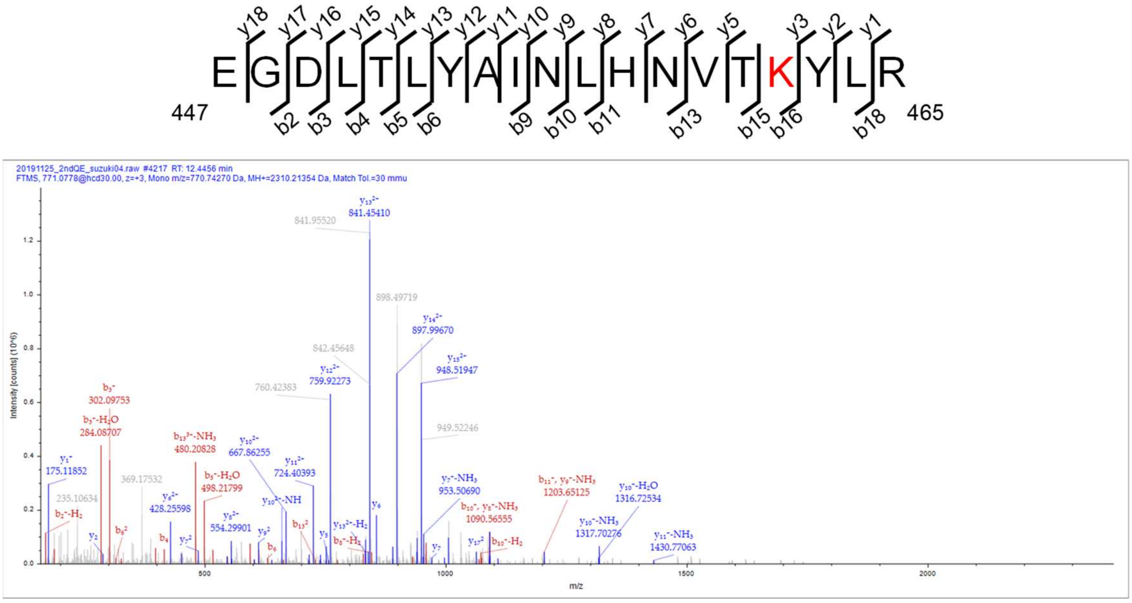

#1	b⁺	b²⁺	b³⁺	Seq.	y⁺	y²⁺	y³⁺	#2
1	130.04987	65.52857	44.02147	E				19
2	187.07133	94.03930	63.02863	G	2181.17027	1091.08877	727.72827	18
3	302.09828	151.55278	101.37094	D	2124.14880	1062.57804	708.72112	17
4	415.18234	208.09481	139.06563	L	2009.12186	1005.06457	670.37880	16
5	516.23002	258.61865	172.74819	T	1896.03779	948.52254	632.68412	15
6	629.31408	315.16068	210.44288	L	1794.99012	897.99870	599.00156	14
7	792.37741	396.69234	264.79732	Y	1681.90605	841.45666	561.30687	13
8	863.41452	432.21090	288.47636	A	1518.84272	759.92500	506.95243	12
9	976.49859	488.75293	326.17105	I	1447.80561	724.40644	483.27339	11
10	1090.54152	545.77440	364.18536	N	1334.72155	667.86441	445.57870	10
11	1203.62558	602.31643	401.88004	L	1220.67862	610.84295	407.56439	9
12	1340.68449	670.84588	447.56635	H	1107.59456	554.30092	369.86970	8
13	1455.71144	728.35936	485.90866	N-Deamidated	970.53564	485.77146	324.18340	7
14	1554.77985	777.89356	518.93147	V	855.50870	428.25799	285.84108	6
15	1655.82753	828.41740	552.61403	T	756.44029	378.72378	252.81828	5
16	1859.95379	930.48053	620.65611	K-Delta:H(4)C(6)	655.39261	328.19994	219.13572	4
17	2023.01712	1012.01220	675.01056	Y	451.26634	226.13681	151.09363	3
18	2136.10118	1068.55423	712.70525	L	288.20302	144.60515	96.73919	2
19				R	175.11895	88.06311	59.04450	1

Supplementary Figure 4-P

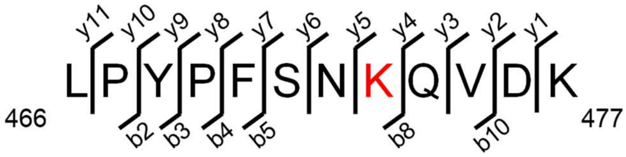

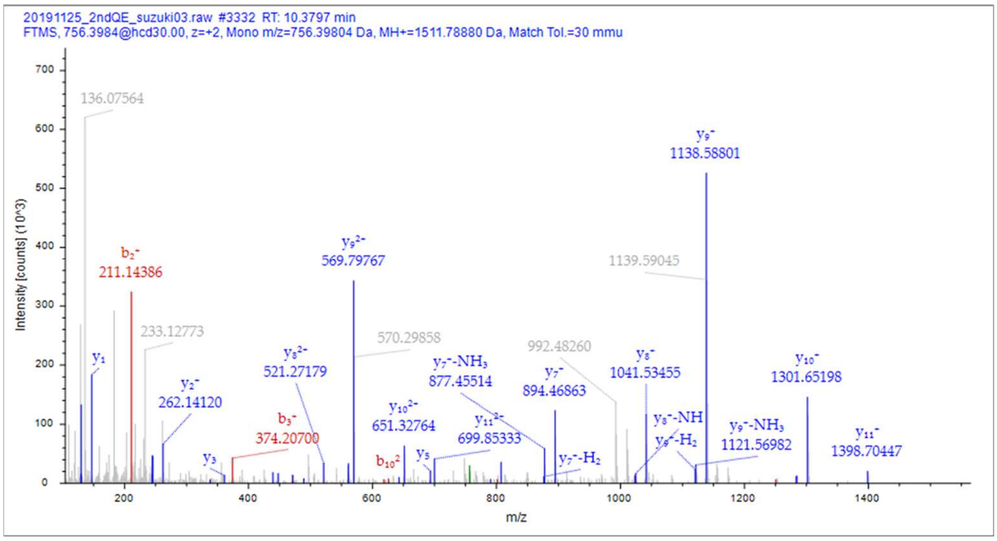

#1	b⁺	b²⁺	Seq.	y⁺	y²⁺	#2
1	114.09134	57.54931	L			12
2	211.14410	106.07569	P	1398.70523	699.85625	11
3	374.20743	187.60735	Y	1301.65246	651.32987	10
4	471.26020	236.13374	P	1138.58913	569.79821	9
5	618.32861	309.66794	F	1041.53637	521.27182	8
6	705.36064	353.18396	S	894.46796	447.73762	7
7	819.40357	410.20542	N	807.43593	404.22160	6
8	1023.52983	512.26855	K-Delta:H(4)C(6)	693.39300	347.20014	5
9	1151.58841	576.29784	Q	489.26674	245.13701	4
10	1250.65682	625.83205	V	361.20816	181.10772	3
11	1365.68376	683.34552	D	262.13975	131.57351	2
12			K	147.11280	74.06004	1

Supplementary Figure 4-Q

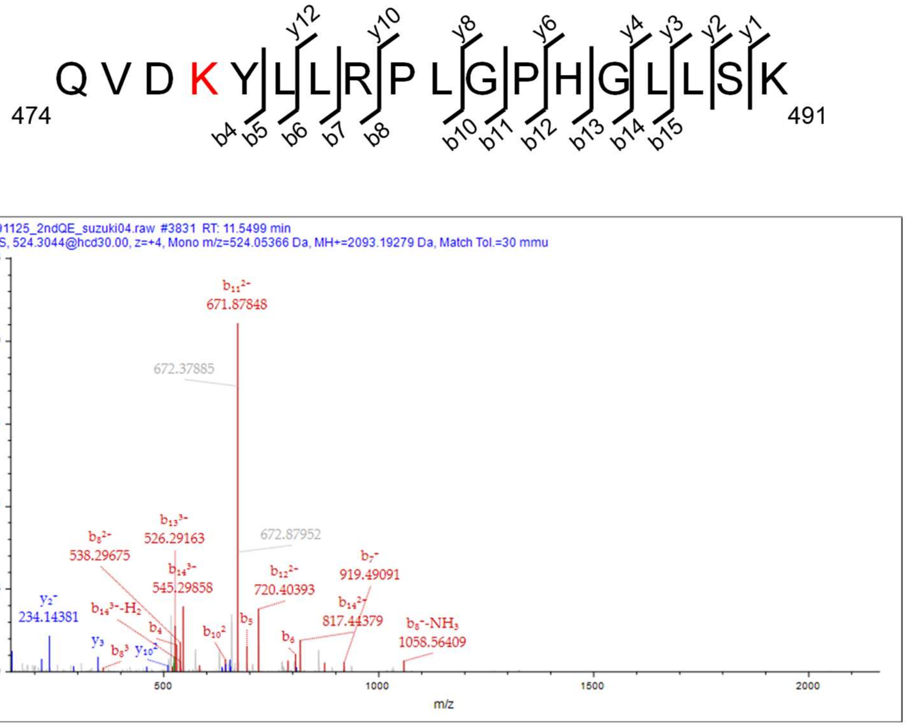

#1	b⁺	b²⁺	b³⁺	b⁴⁺	Seq.	y⁺	y²⁺	y³⁺	y⁴⁺	#2
1	112.03931	56.52329	38.01795	28.76528	Q-Gln->pyro-Glu					18
2	211.10772	106.05750	71.04076	53.53239	V	1982.15858	991.58293	661.39104	496.29510	17
3	326.13466	163.57097	109.38307	82.28912	D	1883.09016	942.04872	628.36824	471.52800	16
4	530.26092	265.63410	177.42516	133.32069	K-Delta:H(4)C(6)	1768.06322	884.53525	590.02592	442.77126	15
5	693.32425	347.16576	231.77960	174.08652	Y	1563.93696	782.47212	521.98384	391.73970	14
6	806.40832	403.70780	269.47429	202.35754	L	1400.87363	700.94045	467.62939	350.97386	13
7	919.49238	460.24983	307.16898	230.62855	L	1287.78957	644.39842	429.93471	322.70285	12
8	1075.59349	538.30038	359.20268	269.65383	R	1174.70550	587.85639	392.24002	294.43183	11
9	1172.64626	586.82677	391.55360	293.91702	P	1018.60439	509.80583	340.20631	255.40656	10
10	1285.73032	643.36880	429.24829	322.18804	L	921.55163	461.27945	307.85539	231.14336	9
11	1342.75178	671.87953	448.25545	336.44340	G	808.46756	404.73742	270.16071	202.87235	8
12	1439.80455	720.40591	480.60637	360.70659	P	751.44610	376.22669	251.15355	188.61698	7
13	1576.86346	788.93537	526.29267	394.97132	H	654.39334	327.70031	218.80263	164.35379	6
14	1633.88492	817.44610	545.29983	409.22669	G	517.33442	259.17085	173.11633	130.08906	5
15	1746.96899	873.98813	582.99451	437.49770	L	460.31296	230.66012	154.10917	115.83370	4
16	1860.05305	930.53016	620.68920	465.76872	L	347.22890	174.11809	116.41448	87.56268	3
17	1947.08508	974.04618	649.69988	487.52673	S	234.14483	117.57605	78.71980	59.29167	2
18					K	147.11280	74.06004	49.70912	37.53366	1

Supplementary Figure 4. MS/MS spectra of peptides containing ACR-modified amino acids of proHPSE.

Supplementary Table 1: Identified peptide sequences with ACR addition.

Position	Peptide sequence	Modifications	m/z (Da)	Charge	MH⁺ (Da)	Theoretical MH⁺ (Da)	Ions score
71-81	FLILLGSPKLR	1xDelta:H(4)C(6) [K9]	444.95168	3	1332.84048	1332.8402	18
99-108	TDFLIFDPKK	1xDelta:H(4)C(6) [K]	433.90378	3	1299.69677	1299.69835	28
108-115	KESTFEER	1xDelta:H(4)C(6) [K1]	551.26512	2	1101.52296	1101.52111	30
129-141	YGSIPPDVEEKLR	1xDelta:H(4)C(6) [K11]	526.94135	3	1578.80949	1578.81623	34
160-167	FKNSTYSR	1xDeamidated [N3]; 1xDelta:H(4)C(6) [K2]	360.5094	3	1079.51364	1079.51564	28
194-214	TADLQWNSSNAQLLLDYCSSK	1xDeamidated [N]; 1xAcrolein adduct2 [N-Term]	1198.55965	2	2396.11203	2396.10747	87
215-232	GYNISWELGNEPNSFLKK	1xDeamidated [N]; 1xDelta:H(4)C(6) [K]	725.02474	3	2173.05966	2173.06005	47
232-251	KADIFINGSQLGEDFIQLHK	1xDeamidated [Q/N]; 1xDelta:H(4)C(6) [K1]	784.07118	3	2350.19897	2350.20777	63
233-254	ADIFINGSQLGEDFIQLHKLLR	1xDeamidated [N/Q]; 1xDelta:H(4)C(6) [K19]	651.8523	4	2604.38739	2604.38205	30
281-303	SFLKAGGEVIDSVTWHHYYLNGR	1xDeamidated [N21]; 1xDelta:H(4)C(6) [K4]	682.34141	4	2726.34382	2726.33616	35
308-338	EDFLNPDVLDIFISSVQKVFQVVESTRPGKK	1xDelta:H(4)C(6) [K18]	722.98902	5	3610.91599	3610.91556	29
412-417	KLVGTK	1xDelta:H(4)C(6) [K1]	361.23349	2	721.45971	721.46069	14

418-428	VLMASVQGSKR	1xOxidation [M3];<br>1xDelta:H(4)C(6) [K10]	423.23214	3	1267.68188	1267.68272	26
433-444	VYLHCTNTDNPR	1xAcrolein adduct2 [N-Term]	735.84497	2	1470.68266	1470.67942	66
445-462	YKEGDLTLYAINLHNVTK	1xDeamidated [N15];<br>1xDelta:H(4)C(6) [K2]	543.03565	4	2169.12079	2169.12265	29
447-465	EGDLTLYAINLHNVTKYLR	1xDeamidated [N13];<br>1xDelta:H(4)C(6) [K16]	770.7427	3	2310.21354	2310.21286	30
466-477	LPYPFSNKQVDK	1xDelta:H(4)C(6) [K8]	756.39804	2	1511.7888	1511.78929	35
474-491	QVDKYLLRPLGPHGLLSK	1xGln->pyro-Glu [N-Term];<br>1xDelta:H(4)C(6) [K4]	524.05366	4	2093.19279	2093.19061	19

Supplementary Table 2: List of antibodies and reagent.

Antibody or reagent	Supplier	No.	Species	Dilution
HPSE1 (H-80)	SANTA CRUZ BIOTECHNOLOGY	sc25825	Polyclonal rabbit	1: 2000
HPSE1	InSight	INS-26-2-0000-11	Polyclonal rabbit	1: 2000
MMP-9	R&D System	AB911	Polyclonal goat	1: 2000
HYAL1	SANTA CRUZ BIOTECHNOLOGY	sc101340	Monoclonal mouse	1: 2000
HYAL2	MyBioSource	MBS2520332	Polyclonal rabbit	1: 2000
HYAL4	SANTA CRUZ BIOTECHNOLOGY	sc-377369	Monoclonal mouse	1: 2000
FDP-Lys	Nichiyu	—	Monoclonal mouse	1: 1000
β-actin	abcam	ab8226	Monoclonal mouse	1: 4000
Isolectin GS-IB4 from *griffonia simplicfolia*, Alexa Fluor® 647 conjugate	ThermoFisher Scientific	I32450	*Griffonia simplicfolia*	1:50
Donkey anti-Rabbit IgG (H+L) Highly Cross-Adsorbed Secondary Antibody, Alexa Fluor® 488	ThermoFisher Scientific	A21206	Polyclonal donkey	1:200
-Cellstain®-DAPI solution	Dojindo	D523		
